# The Ground Beetle *Poecilus* (Carabidae) Gut Microbiome and Its Functionality

**DOI:** 10.1007/s00248-025-02579-0

**Published:** 2025-07-30

**Authors:** Chiara Braglia, Simone Cutajar, Serena Magagnoli, Diana Asciano, Giovanni Burgio, Diana Di Gioia, Loredana Baffoni, Daniele Alberoni

**Affiliations:** 1 Dipartimento di Scienze e Tecnologie Agro-Alimentari (DISTAL), University of Bologna, Viale Fanin 42, 40127 Bologna, Italy; 2 Institute of Earth Systems, L-Università tà Malta, Msida, MSD 2080 Malta

**Keywords:** *Carnobacterium*, *Gilliamella*, *Weissella*, *Nosema ceranae*, Agroecosystems, Mutual-Information filter

## Abstract

Ground beetles of the genus *Poecilus* (Carabidae) play key ecological roles in pest control and soil health. However, their gut microbiome remains largely unexplored despite increasing interest in insect-associated microbiota and its environmental implications. This study used next-generation sequencing and qPCR to characterise the gut microbiome of *Poecilus* beetles collected from organic and conventional tomato fields. Core microbiota were identified through prevalence–abundance filtering, revealing dominant genera including *Gilliamella*, *Weissella*, *Enterobacter*, and *Enterococcus*, alongside several low-abundance but consistently present taxa. Notably, *Carnobacterium* was detected for the first time in an insect host, and *Nosema ceranae* was identified for the first time in Carabidae, expanding the known host range of this microsporidian pathogen. Functional predictions based on 16S rRNA data and comparative genomic analysis showed enrichment in pathways related to amino acid synthesis, protein degradation, and monosaccharide metabolism. Significant inter-individual variation in microbial diversity and predicted functionality was observed, with lowest diversity and metabolic potential in beetles from conventionally managed fields, potentially indicating dysbiosis and environmental stress. The detection of *Nosema* and *Serratia* pathogens in some individuals adds new insights into pathogen dynamics within carabid beetles. Our findings reveal that the gut microbiome of *Poecilus* may be shaped by environmental factors and agricultural practices, influencing host health and ecological performance. These insights support the use of *Poecilus* as a bioindicator for soil ecosystem health and highlight the potential of microbiome-based metrics in agroecological monitoring.

**Supplementary Information:**

The online version contains supplementary material available at 10.1007/s00248-025-02579-0.

## Introduction

*Poecilus* (Carabidae) is recognised as a key ground beetle genus that helps maintain ecological balance in various ecosystems [1]. These beetles, like many other carabids, are important predators of agricultural pests, such as aphids, caterpillars, and slugs, naturally reducing pest populations and the reliance on chemical pesticides [2–4]. For example, *Poecilus cupreus* has been shown to prey on slugs and their eggs, suggesting a direct role in managing slug populations [5]. Beyond their pest-control role, *Poecilus* species may also contribute to soil health by aiding in the decomposition of organic matter and facilitating nutrient cycling, a function shared broadly among carabids [6, 7]. The ability of *Poecilus* to inhabit diverse agricultural habitats, including cereal fields and field margins, enhances their utility in integrated pest management strategies across various agricultural landscapes [8].

The presence of *Poecilus* beetles in an ecosystem often serves as an indicator of environmental health and biodiversity, highlighting the importance of conserving these beetles and their habitats to sustain ecosystem services [4, 9, 10]. However, their sensitivity to environmental disturbances, such as exposure to pesticides (pyrethroids, fungicides, weed-killers) and heavy metals, negatively impacts their development, metabolism, and the immune function, as documented in other carabids like *Calathus fuscipes* and *Harpalus rufipes* [11–14].

Investigating the gut microbiome of *Poecilus* is especially relevant because insect gut symbionts have long been recognised as vital for nutrition, immune modulation, and overall fitness [15–17]. Dysbiosis in these gut communities is detrimental to insect health [18, 19], while shifts in microbiome composition due to farm management practices [20] and dietary influences [21, 22] have already been demonstrated in other Carabidae, typically characterised by microbial phyla such as *Weissella*, *Spiroplasma*, *Burkholderia*, *Enterobacter*, and *Serratia* [23, 24].

Exploring the gut microbiome of *Poecilus*, a potential biological control candidate, can reveal how environmental and agricultural factors influence beetle health and ecosystem interactions. To date, no studies have characterised the gut microbiome of *Poecilus*, nor examined its potential as a bioindicator of soil health across farming systems. This study aims to fill these gaps by using next-generation sequencing (NGS) to profile the microbes inhabiting* Poecilus* guts and to predict their functional potential. Specifically, this research will elucidate, for the first time, how the core gut microbiome may support beetle health and aid digestion.

## Methods

### Experimental Design and Carabidae Collection

The study was conducted at the Council for Agricultural Research and Economics – Research Centre for Vegetable and Ornamental Crops (CREA-OF) in Monsampolo del Tronto, Marche Region, Italy (latitude 42°53′ N, longitude 13°48′ E), following the same experimental design described in Magagnoli et al., [20]. Briefly, Carabidae were collected from two experimental tomato fields (*Solanum lycopersicum* L., variety SAAB-CRA), one under organic management (ORG), and the other under conventional management (CNV), from early June to late August. Carabid beetles were sampled with standard pitfall traps, 16 cm deep. Each trap consisted of two nested plastic cups filled with commercial vinegar [25]. Following the methodology of Magagnoli et al. [26], five sampling stations were established in each field (ORG and CNV). Each station held two double pitfall traps, for a total of 10 double traps per sampling occasion and management system. Stations were arranged on an offset grid between crop rows: three located within the field interior and two positioned along the outer edge. To prevent rainwater accumulation and minimise bycatch, traps were protected by plastic saucers.

Sampling was conducted on two occasions, on 26 June and 7 August 2019. On each date, pitfall traps were set in the late afternoon and were left overnight; traps were then checked and emptied early the following morning (between 8:00 and 9:00). Collected Carabids were placed in single plastic vials, transported to the laboratory, and immediately frozen (− 80 °C) until further analysis.

### Carabid Dissection, DNA Extraction from Gut, and qPCR Analysis

Before the gut extraction, Carabid samples were processed as described by Magagnoli et al. [20]. Seventeen individuals, 15 from ORG and two from the CNV managed field, were surface-sterilised by immersion in 70% ethanol, followed by two rinses in sterile Ringer’s solution (3 mM CaCl_2_, 182 mM KCl, 46 mM NaCl, 10 mM Tris base; pH 7.2). The entire gut (including foregut, midgut and hindgut) was extracted and clamped with microvascular clamps (1.0–2.5 mm, S&T AG, Neuhausen am Rheinfall, Switzerland) and then externally disinfected in ethanol (70%), washed in phosphate buffered saline (PBS; pH 7.2), placed in sterile 1.5 mL tubes and stored at − 80 °C.

DNA was extracted individually from each dissected gut using the Quick-DNA Tissue/Insect Microprep Kit (Zymo Research, Irvine, CA, USA) following manufacturer’s instructions, after mechanical and chemical lysis. The extracted DNA was quantified and checked for purity using a spectrophotometric method (Infinite® 200; TECAN, Männedorf, Switzerland), and stored at − 20 °C until use. qPCR on total bacteria (eubacteria), *Bartonella*, *Bombilactobacillus* (Firm-4), *Lactobacillus* (Firm-5), *Bifidobacterium*, *Spiroplasma*, *Snodgrassella*, *Gilliamella*, *Enterobacter*, *Serratia*, and *Nosema ceranae* were carried out using the QuantStudio® 5 Real-Time PCR System (Applied Biosystems). Primers used are reported in Table S1. The amplification reactions were performed in a total volume of 10 μL with SYBR Green chemistry, according to Braglia et al. [27]. Each reaction consisted of 5 μL of Fast SYBR Green Master Mix (Applied Biosystem), 0.3 μL of each primer, and 1 μL of DNA and water up to the final volume of 10 μL. Amplification reactions were conducted on the basis of the following protocol: activation at 95 °C for 30 s, 40 cycles of denaturation at 95 °C and annealing from 58.0 to 61.0 °C, according to each target considered, and final melting curve with a temperature gradient increase of 0.3 °C from 60 to 95 °C. Data were expressed as “spores/gut” for *Nosema*, “*luxS* gene copies/gut” for *Serratia*, and “16S rRNA copies/intestine” for the other targets. qPCR results were analysed using RStudio (v 4.3.3, R foundation for Statistical Computing; Vienna, Austria), and bar charts with absolute abundances were generated using ggplot2, dplyr, tidyr, and RColorBrewer packages. The Shapiro–Wilk [28] test and Levene test [29] were used to assess data distribution and homoscedasticity. Kendall test (more rigorous with low samples number) was used to assess correlations and distribution between *Spiroplasma* versus *Nosema* and *Serratia* using ggplot2, dplyr, ggrepel, and ggpubr packages.

### Amplicon Based NGS Sequencing

The V3–V4 region of the 16S rRNA gene of the Carabids’ microbiome was sequenced according to Magagnoli et al. [20]. The V3–V4 region was amplified (primers reported in Table S1), using HiFi KAPA HiFi HotStart ReadyMix (KAPA Biosystem, Woburn, MA, USA). The PCR products were purified using the AMPure beads XP purification system (Backman Coulter, UK) following the instructions for Illumina 16S ribosomial RNA Gene Amplicon. Amplicons were then barcoded using the Nextera XT v2 Index Kit D (Illumina, San Diego, CA, USA) following manufacturer’s instructions, and then purified with AMPure beads XP purification system protocol (Beckman Coulter, UK). Library quantification was carried out with Qubit 2.0 Fluorometer (Invitrogen, Life Technologies, Carlsbad, CA, USA). Finally, libraries were sequenced on the MiSeq platform (2 × 300 pair-end sequencing) by Bio-Fab Research s.r.l. (Rome, Italy) using V3 chemistry.

### Bioinformatics Analysis of NGS Data

The raw sequencing reads obtained from the MiSeq platform were processed using QIIME 1.9 [30], according to Magagnoli et al. [20]. Sequence demultiplexing was conducted with validate_mapping_file.py to ensure accurate sample metadata. Paired-end reads were merged using join_paired_ends.py, and unmerged sequences were discarded. After merging, sequences were quality-filtered using the multiple_split_libraries_fastq.py script. Chimeric sequences were identified using USEARCH v6.1 through the identify_chimeric_seqs.py script, using the SILVA 128 database as reference. Identified chimeras were subsequently removed with filter_fasta.py. Operational taxonomic units (OTUs) were clustered using USEARCH v6.1 via the pick_otus.py script. Representative sequences were selected with pick_rep_set.py, using the most abundant sequence as the representative for each OTU. Taxonomic classification was performed with the RDP Classifier through the assign_taxonomy.py script. Sequences were aligned using align_seqs.py, and the phylogenetic tree was constructed with make_phylogeny.py. An OTU table was generated using make_otu_table.py, and rarefaction was applied while performing core diversity analyses using core_diversity_analyses.py. Taxonomic summaries were generated using summarise_taxa.py, and heatmaps were created with make_otu_heatmap.py. OTU table was pruned to remove low-abundance taxa (filter_otus_from_otu_table.py), ensuring robust downstream analysis.

### Data Filtering and Core Gut Microbiota Definition

To ensure data quality and improve the accuracy of microbial community analyses, raw sequencing data underwent a structured pre-processing workflow.

After an initial abundance-threshold filter, we refined the OTU table with a mutual-information (MI) filter (following Mokhtari, Ridenhour [31]) to avoid arbitrary cut-offs, suppress noise, and exclude uninformative taxa while retaining meaningful microbial associations. The filtered table was then used for all subsequent diversity and network analyses. Taxa with MI below a predefined threshold (τ) were considered uninformative and excluded from downstream analysis. The MI filtering process was carried out using the infotheo package in R, while the microbial association network was constructed using the igraph package, and the optimised threshold (*τ*) was determined based on MI retention analysis. We selected *τ* = 0.01 based on exploratory comparisons across a range of candidate thresholds (*τ* = 0.005, 0.01, 0.02, 0.05). Thresholds below 0.01 (e.g., 0.005) retained a large number of nearly ubiquitous or rare OTUs with minimal discriminatory power, while higher thresholds (e.g., > 0.02) resulted in excessive OTU loss, reducing the diversity captured in the data. The chosen threshold (*τ* = 0.01) retained OTUs that contributed > 90% of cumulative relative abundance while substantially reducing dimensionality, allowing us to preserve the informative structure of the data while minimising noise. To support this choice, we evaluated the impact of different *τ* values on (i) the number of OTUs retained, (ii) their cumulative relative abundance, and (iii) their contribution to sample separation in principal coordinate analysis (PCoA). *τ* = 0.01 provided a clear balance between information retention and noise reduction and was therefore used as a conservative yet effective cutoff for filtering.

Following this step, 210 OTUs were removed, and the final OTU table contained 327 OTUs across 17 samples, ensuring a balance between data retention and artefact removal. The total information loss after MI filtering was 4.25%, indicating that the method effectively reduced noise while preserving the dataset’s biological integrity.

Following the pre-processing and MI-based (﻿mutual information﻿-based) filtering of raw sequencing data, raw taxon-level read counts derived from 16S rRNA gene amplicon sequencing were adjusted to account for gene copy number variation. To obtain absolute abundance estimates, relative abundances (0–1) were multiplied by the total bacterial load per sample as measured by qPCR targeting universal total bacteria 16S rRNA genes. The core gut microbiota of *Poecilus* was subsequently defined using a combined prevalence-abundance framework, applied across multiple taxonomic ranks. Core members were identified based on two conservative criteria using microbiome R package; a prevalence threshold of ≥ 80% (present in at least 80% of samples) and a mean relative abundance threshold of ≥ 0.01% across all samples. Further information on the NGS data analysis procedures and management are reported in the Appendix I: NGS data analysis and management.

### Gut Microbiome Functionality Assessment and Prediction

We used the core gut microbial taxa identified by 16S rRNA next-generation sequencing (NGS) to predict the functional profiles of each *Poecilus* individual’s gut microbiome. Fully sequenced genomes of type strain bacterial taxa available in NCBI GenBank were functionally annotated using RAST (SEED Viewer version 2.0) [32, 33] and the KEGG orthology database [34]. The analysis focused on metabolic pathways related to (i) vitamins, nitrogen, monosaccharides, hormones and aromatic compounds metabolisms; (ii) polysaccharides, protein, and alkaloids degradation; (iii) amino acids and fatty acids biosynthesis; and (iv) antibiotics and toxic compounds’ resistance. The specific pathways considered for each class are reported in Table S2. Each microbial strain was screened for the presence or absence of the selected pathways. We predicted the functional potential of each *Poecilus* individual’s gut microbiome using the relative abundances of microbial genera obtained from NGS. Specifically, core gut microbiome taxa relative abundance was multiplied by the estimated functional capacity for each specific genus. To predict and represent the microbiota functional distribution for each *Poecilus* specimen, the functional absolute values were converted into relative values for each microbial taxon and expressed as a “predicted score value”. Moreover, to highlight active pathways, EC numbers were mapped to KEGG pathways using the KEGG REST API (https://www.kegg.jp/kegg/rest/) and the results were applied to the abundance of each sampled individual. Functionality assessment and prediction were performed with RStudio (v 4.3.3) using tidyverse and pheatmap packages.

### Statistical Analysis

Statistical analysis for qPCR and NGS data (α-diversity and taxon analysis) was performed with the RStudio (v. 4.3.3), according to Baffoni et al. [35]. β–diversity index was based on the QIIME statistical elaboration reports, and permutational analysis of variance (PERMANOVA) was conducted for statistical significance. Graphs were generated with ggplot2 and ggpubr. A comparison of the concentration of eubacteria concentration between each farm management (ORG against CNV) was employed using the non-parametric median test.

To investigate whether gut microbial functionality was associated with microbiota diversity, we conducted a correlation analysis between the relative abundances of predicted functional pathways and microbial diversity metrics across *Poecilus* beetle samples. Functional abundances (e.g., amino acid synthesis, monosaccharide metabolism) were derived from metagenomic predictions, while diversity was quantified using three standard indices: Shannon diversity, Simpson diversity, and observed richness (taxonomic count).

We calculated Spearman’s rank correlation coefficient (*ρ*) between individual functional categories and diversity values across 17 beetle gut microbiota samples for each diversity metric. *P*-values were adjusted for multiple comparisons using the Benjamini–Hochberg false discovery rate (FDR) correction.

## Results

A total of 17 *Poecilus* specimens were collected, with 15 specimens collected from organically managed tomato fields (ORG) and two specimens collected in from conventionally managed tomato fields (CNV).

### Next-Generation Sequencing (NGS) Output Results

High-throughput sequencing of *Poecilus* gut samples yielded a total of 1,176,300 raw sequences, prior to chimera removal. After chimera filtering, 1,144,200 sequences were retained, and further length filtering (removal of sequences < 300 bp) resulted in 1,137,645 high-quality sequences. These were used as the input for downstream analysis.

Microbial community processing was conducted using QIIME 1.9.1. OTU picking was performed with USEARCH61, producing 7,827 OTUs. Taxonomic classification was assigned using the UCLUST algorithm against the SILVA 128 database. To ensure robust comparisons across samples, the data were rarefied to the minimum sequencing depth observed across the dataset, 29,611 reads per sample. This rarefaction depth preserved all 17 samples for downstream diversity and taxonomic analyses. Post-rarefaction, the dataset had a mean read count of 66,920 sequences per sample (range: 29,611 to 102,067), with a standard deviation of 19,935 reads.

### Alpha and Beta Diversity of Gut Microbiota Across Poecilus Samples

Alpha diversity analysis revealed substantial variation in the within-sample diversity of gut microbiota across *Poecilus* individuals (Table S3). Shannon diversity indices ranged from 0.73 for *Poecilus* 1 (P1) to 1.89 for *Poecilus* 17 (P17), indicating a broad spectrum of community complexity. Sample P1, collected from a conventionally managed field (CNV), exhibited the lowest diversity, suggesting dominance by one or a few highly abundant taxa, particularly *Spiroplasma* at 99.1% abundance. Conversely, sample P17, collected from an organically managed field (ORG), harboured a more evenly distributed and diverse microbial community. Sample P1 showed the lowest Zahl Shannon (ZS) diversity across all metrics (ZS = 0.0077; Simpson = 0.50), due to *Spiroplasma* dominance (99.1% of reads). In contrast, P15 was the most diverse (ZS = 2.37), suggesting a rich and balanced microbiota, followed by P17 (ZS = 2.04; Simpson = 0.71). Most samples had intermediate ZS values e.g., P4, P5, P8, and P2 (1.7–1.9), indicating moderate diversity. Lower-diversity samples like P11 (ZS = 0.72) and P9 (ZS = 0.98) may reflect reduced complexity. Simpson indices confirmed these trends, with high values also seen in P12, P15, and P4. Taxonomic richness (observed OTUs) varied markedly, from 49 in P9 to 184 in P4 and 174 in P12. Sample P4 combined high richness with moderate diversity, indicating many low-abundance taxa. In contrast, P1 hosted 79 OTUs showing the lowest diversity, reflecting a highly uneven microbiota. Overall, both classic and unbiased estimators highlight strong inter-individual variation, from mono-dominant to rich, and balanced gut communities. Beta diversity was assessed using Bray–Curtis dissimilarity followed by principal coordinate analysis (PCoA) to visualise differences in microbial community composition across samples (Figure S1). A PERMANOVA on the same matrix detected no significant compositional differences among groups (*p* > 0.05). Nevertheless, the PCoA plot revealed distinct clustering patterns, indicating variation in microbial communities between samples. Notably, samples such as P1, P2, P11, and P12 appeared more separated from the main cluster along the first principal coordinate (PC1), suggesting they harboured more unique microbial communities compared to the rest. In contrast, samples like P4, P5, P3, and P17 formed a tighter group, indicating higher similarity in community composition. The spread of points across the ordination space reflects a gradient of dissimilarity, demonstrating that microbial communities vary across samples.

### Core Gut Microbiota Composition

At the phylum level, the gut microbiota of *Poecilus* was dominated by Pseudomonadota (basionym Proteobacteria; mean relative abundance = 52.3%), followed by Bacillota (basionym Firmicutes; 23.3%), Actinomycetota (basionym Actinobacteria; 8.6%), and Bacteroidota (basionym Bacteroidetes; 6.7%). These four phyla were consistently detected across all samples (with a prevalence of 1.0), indicating their stable presence in the *Poecilus* gut environment.

Other phyla such as Mycoplasmatota (8.3%), Verrucomicrobiota (0.62%), and Spirochaetota (0.07%) were also present, though with lower consistency and relative abundance. Notably, Tenericutes exhibited a moderate mean abundance but were only detected in about 65% of samples, suggesting possible host- or environment-specific patterns.

Several low-abundance phyla, including Planctomycetota, Saccharimonadota, Tectimicrobiota, Gemmatimonadota, and Nitrospirota, were detected sporadically, often below 0.01% mean abundance, indicating they may represent transient or rare members of the gut microbial community (Table S4).

At the family level (Table S5), Enterobacteriaceae (44.7%) and Orbaceae (30.4%) were dominant and occurred in every sample. Leuconostocaceae, mainly *Weissella* (13.4%) was also frequent found in 94.1% of the samples. Less abundant but consistent families included Staphylococcaceae (1.45%), Carnobacteriaceae (0.356%), Lactobacillaceae (0.294%), and Bacillaceae (0.0398%). These results highlight a core gut microbiota composed of both dominant and stable low-abundance taxa (Figure S2 and S3).

**Fig. 1:**
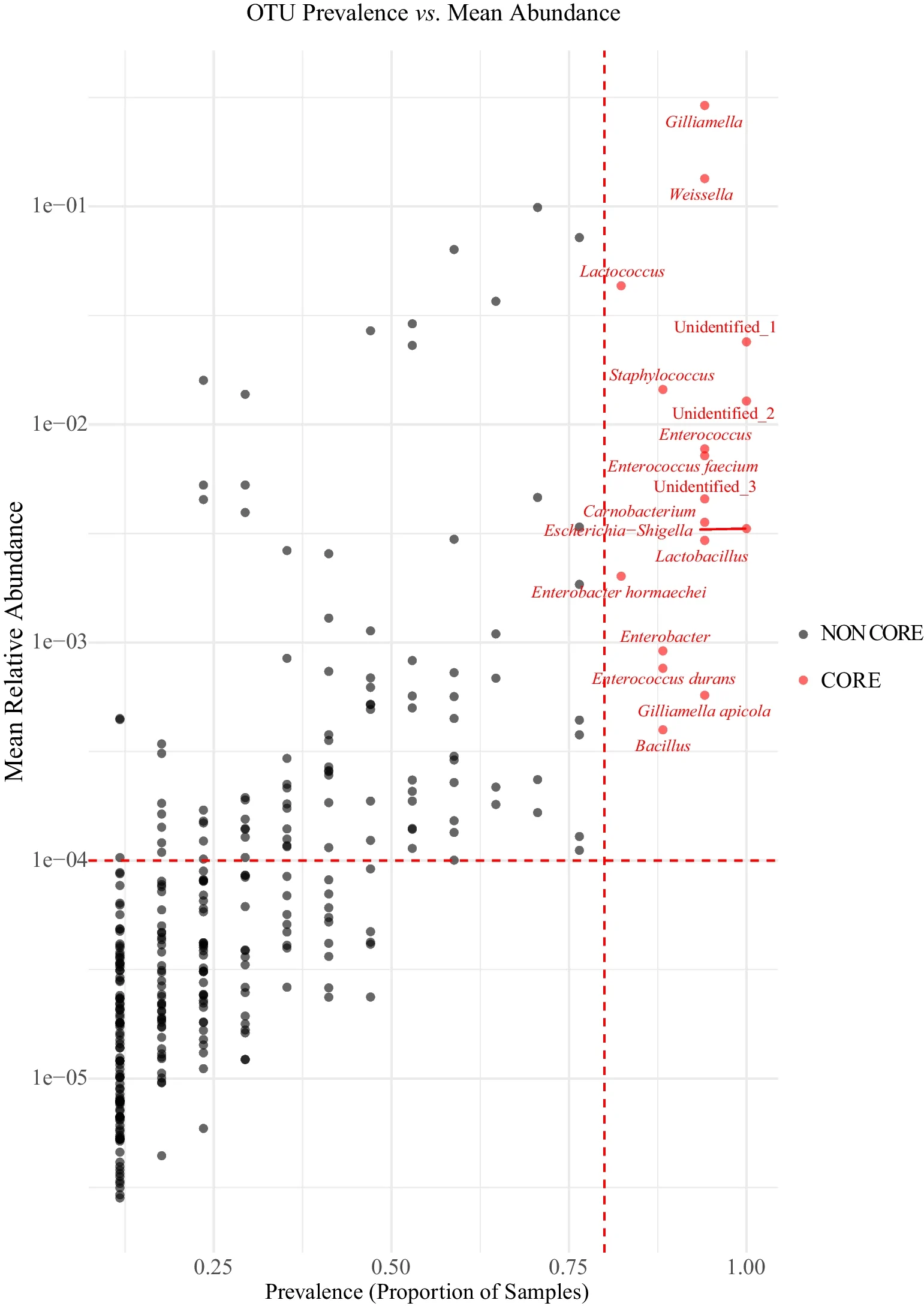
Prevalence–abundance distribution of gut microbiota in *Poecilus* beetles. Scatter plot of operational taxonomic units (OTUs) showing their prevalence (*X*-axis: proportion of individuals) versus mean relative abundance (*Y*-axis: mean relative abundance, log scale). Core taxa (in red) are defined as those present in ≥ 80% of samples and with ≥ 0.01% mean abundance, marked by dashed red thresholds. Most core taxa, such as *Gilliamella* and *Weissella*, are both widespread and relatively abundant, while non-core taxa are generally lower in both metrics. This visualisation distinguishes highly conserved microbial members from rare or sporadic taxa

To define the core gut microbiota of *Poecilus*, OTUs were evaluated using both prevalence and mean relative abundance thresholds. OTUs were considered part of the core microbiota if they were present in at least 80% of samples (prevalence ≥ 0.80) and exhibited a mean relative abundance ≥ 0.01% across all samples. Based on these criteria, 17 OTUs were identified as core taxa (Figure S4 and Table S6). The prevalence–abundance scatter plot (Fig. 1) illustrates this classification, and OTUs exceeding both thresholds are shown in red, distinguishing them from the background of rarer or less abundant taxa. These core taxa represent the most stable and consistently present members of the *Poecilus* gut microbiome.

Out of the 17 core OTUs identified, only five could be resolved to the species level: *Enterococcus durans*, *Enterococcus faecium*, *Enterococcus mundtii*, *Gilliamella apicola*, and *Enterobacter hormaechei*. The remaining OTUs lacked full taxonomic resolution. Twelve were identified to the genus level, including *Bacillus*, *Carnobacterium*, three *Enterobacter* (strains 1, 2 and 3), *Enterococcus*, two *Gilliamella* (strains 1 and 2), *Lactobacillus*, *Lactococcus*, *Staphylococcus*, and *Weissella*. One OTU was assigned only to the family *Enterobacteriaceae* and could not be classified at lower taxonomic ranks. The stacked bar plot (Figure S5) shows the relative abundance of core OTUs across individual samples. While individual variation is evident, several taxa, *Enterobacter* strain 3, *Enterobacteriaceae*, and *Gilliamella* strain 1 (all present in 100% of samples, at 0.33%, 2.39%, and 1.28% relative abundance, respectively); *Gilliamella* strain 2 (94.1% prevalence, with a notably high relative abundance of 29.0%); *Weissella* (94.1% prevalence; 13.4% relative abundance); and *Staphylococcus* (88.2% prevalence; 1.45% relative abundance), appear consistently and in high proportions across multiple individuals, further supporting their designation as core taxa. Conversely, certain samples, such as P1, P11, P4, and P13, show much lower proportions of core taxa (e.g., only 0.0514% of P1’s microbiota consisted of core taxa), suggesting the influence of ecological or host-related factors on microbiome composition. A heatmap of the 17 core genera (Figure S6) shows their relative abundances, with hierarchical clustering highlighting co-occurrence patterns. At the genus level, core taxa included *Enterobacter*, *Gilliamella*, *Weissella*, *Enterococcus*, *Staphylococcus*, *Carnobacterium*, *Bacillus*, and *Enterobacteriaceae*, with four (*Enterobacter* strain 3, *Enterobacteriaceae*, *Gilliamella*, *Weissella*) present in 100% of samples. *Weissella* was notably abundant (13.4%) (Table S7).

### Predicted Functionality Assessment

**Fig. 2:**
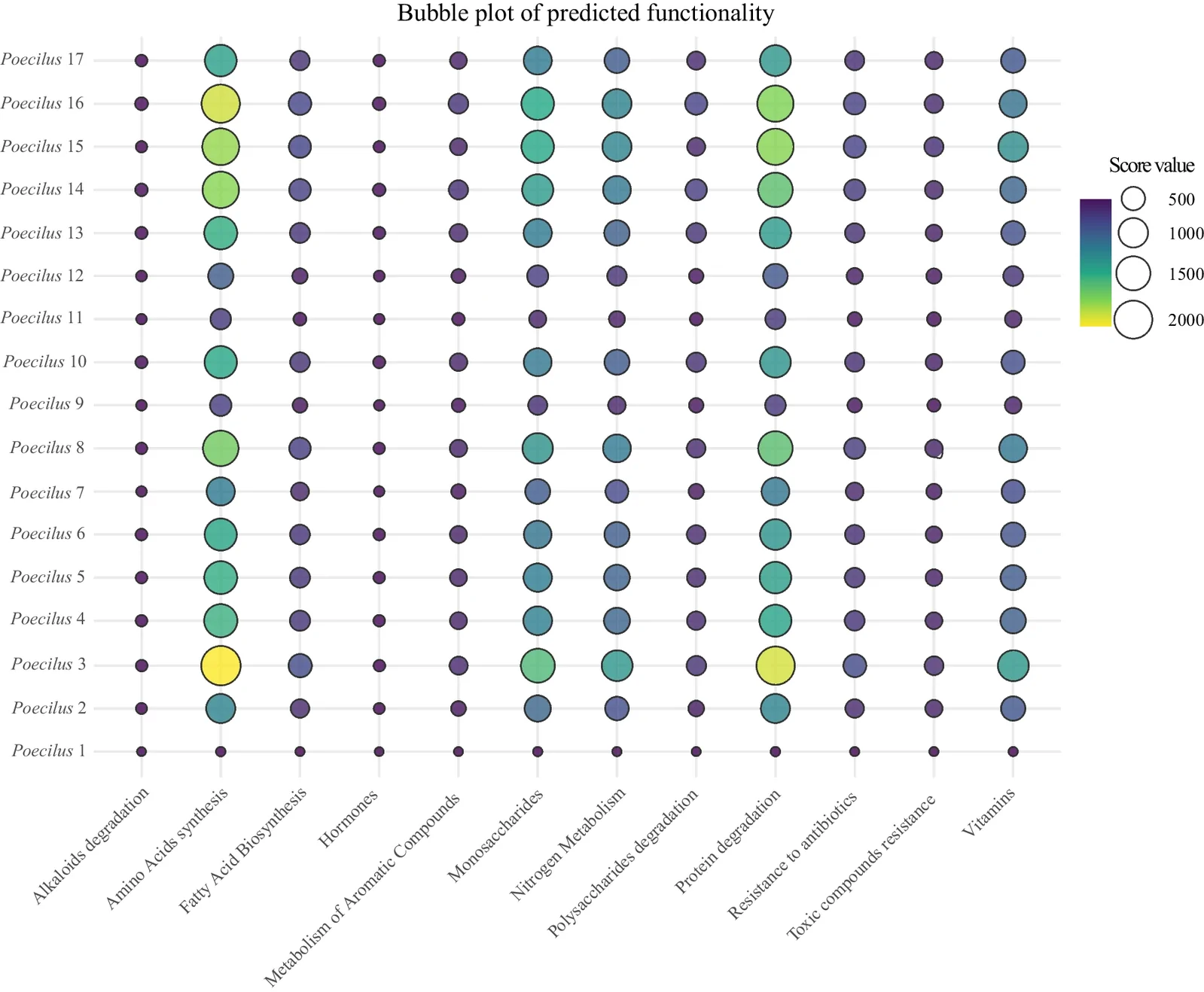
Predicted functional scores derived from the relative abundances of core gut microbiome taxa in each *Poecilus* individual

The functionality prediction assessment on the core gut microbial taxa revealed that protein degradation and amino acid synthesis pathways are the most represented among the main microbial genus and species, followed by monosaccharides, nitrogen, and vitamins metabolisms pathways (Figures S7 and S8). *Enterobacter* was the most performant taxa when the number of pathways highlighted was considered (3525 pathways), followed by *Bacillus* (2162 pathways), and *Enterococcus* (1823 pathways; Table S8). Prediction scores corroborated our functional assessment of the *Poecilus* gut microbiome. Pathways for protein degradation, amino acid biosynthesis, and the metabolism of monosaccharides, nitrogen, and vitamins were the most prominent. In contrast, pathways involved in alkaloid degradation, hormone metabolism, and resistance to toxic compounds received the lowest scores. Functional predictions for individual *Poecilus* specimens showed similar distribution patterns across all functional classes (Fig. 2). However, five specimens, P1, P7, P9, P11, and P12 showed markedly lower predicted functional scores (between 4.24 and 3889.43) than the remaining individuals (between 4506.31 and 9672.85), indicating a substantial reduction in gut microbiome functional potential (Table 1).

**Table 1:** Predicted functional scores for selected pathway classes in each *Poecilus* individual

	Amino acid synthesis	Fatty acid biosynthesis	Monosaccharides	Nitrogen metabolism	Protein degradation	Resistance to antibiotics	Toxic compounds resistance	Vitamins	Alkaloids degradation	Hormones	Metabolism of aromatic compounds	Polysaccharides degradation
*Apilactobacillus*	27	4	10	19	37	3	3	15	0	0	0	0
*Bacillus*	535	55	234	174	579	155	75	177	6	2	86	84
*Carnobacterium*	280	44	211	143	289	59	87	118	4	5	16	11
*Enterobacter*	783	123	562	379	663	233	193	235	12	3	189	150
*Enterococcus*	370	61	407	185	416	77	77	186	2	2	18	22
*Gilliamella*	217	47	136	102	184	41	21	81	4	4	31	46
*Lactobacillus*	142	15	169	97	225	33	42	83	0	1	8	8
*Lactococcus*	183	44	141	98	187	43	37	111	3	3	17	24
*Staphylococcus*	317	35	124	103	264	59	50	129	6	7	30	32
*Weissella*	218	50	150	120	215	49	23	138	0	0	12	12

The analysis of the top 10 predicted enzymatic functions (EC) per sample revealed consistent representation of multiple metabolic categories (Figure S9). Among the most represented functions, enzymes involved in amino acid metabolism were predominant. These included aspartate aminotransferases (EC 2.6.1.1, predicted scores value of 18.3), branched-chain amino acid aminotransferase (EC 2.6.1.42, predicted scores value of 14.7), methionine aminopeptidase (EC 3.4.11.18, predicted score value of 11.5), and diaminopimelate decarboxylase (EC 4.1.1.20, predicted score value of 9.6), which appeared across several individual profiles. Functions involved in protein degradation were also abundant, such as ATP-dependent Clp protease proteolytic subunit (EC 3.4.21.92, predicted score value of 13.9), tripeptide aminopeptidase (EC 3.4.11.4, predicted score value of 10.2), and aminopeptidase S (Leu, Val, Phe, Tyr specific) (EC 3.4.11.24, predicted score value of 7.1). Moreover, multiple samples displayed predicted activity of enzymes linked to nucleotide or cofactor metabolism, such as IMP cyclohydrolase (EC 3.5.4.10, predicted score value of 6.4), Inosine-5′-monophosphate dehydrogenase (EC 1.1.1.205 predicted score value of 5.9), and Inositol-1-monophosphatase (EC 3.1.3.25, predicted score value of 5.1). Finally, a few samples exhibited predicted functions related to sugar and monosaccharide metabolism, including UDP-sugar hydrolase (EC 3.6.1.45, predicted score value of 4.8) and 5′-nucleotidase (EC 3.1.3.5, predicted score value of 3.2). Full annotations for each EC, including predicted scores, are provided in Supplementary File 1.

### qPCR Results on Specific Targets

**Fig. 3:**
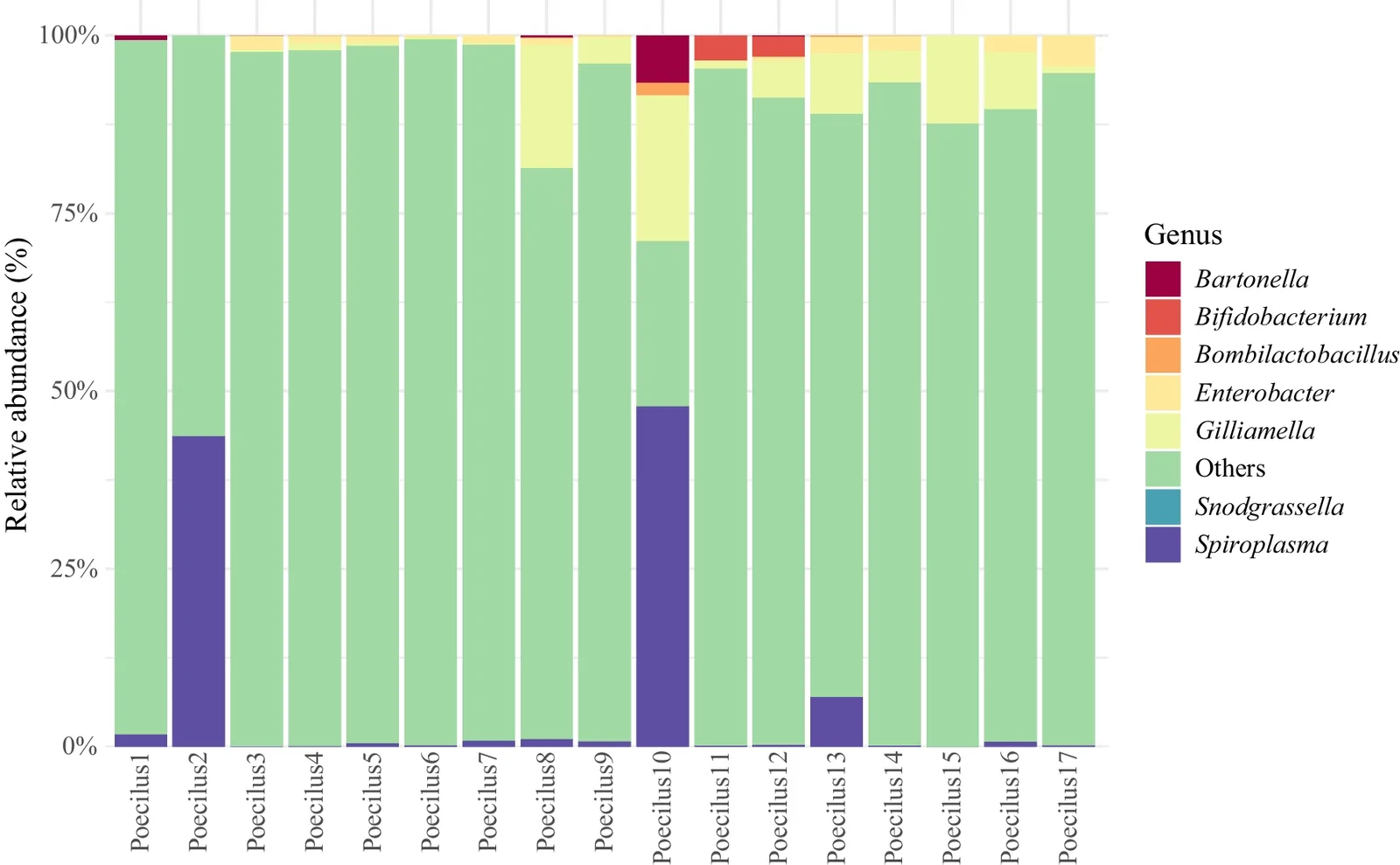
Percentage (absolute abundance) of each qPCR targeted taxon detected in the *Poecilus* gut samples

Quantitative PCR corroborated the NGS results, identifying *Gilliamella* and *Enterobacter* as the dominant gut taxa in *Poecilus* (Fig. 3). *Bifidobacterium* appeared in only five specimens and *Snodgrassella* in two, while *Lactobacillus* (Firm-5) was undetected in all individuals.

**Fig. 4:**
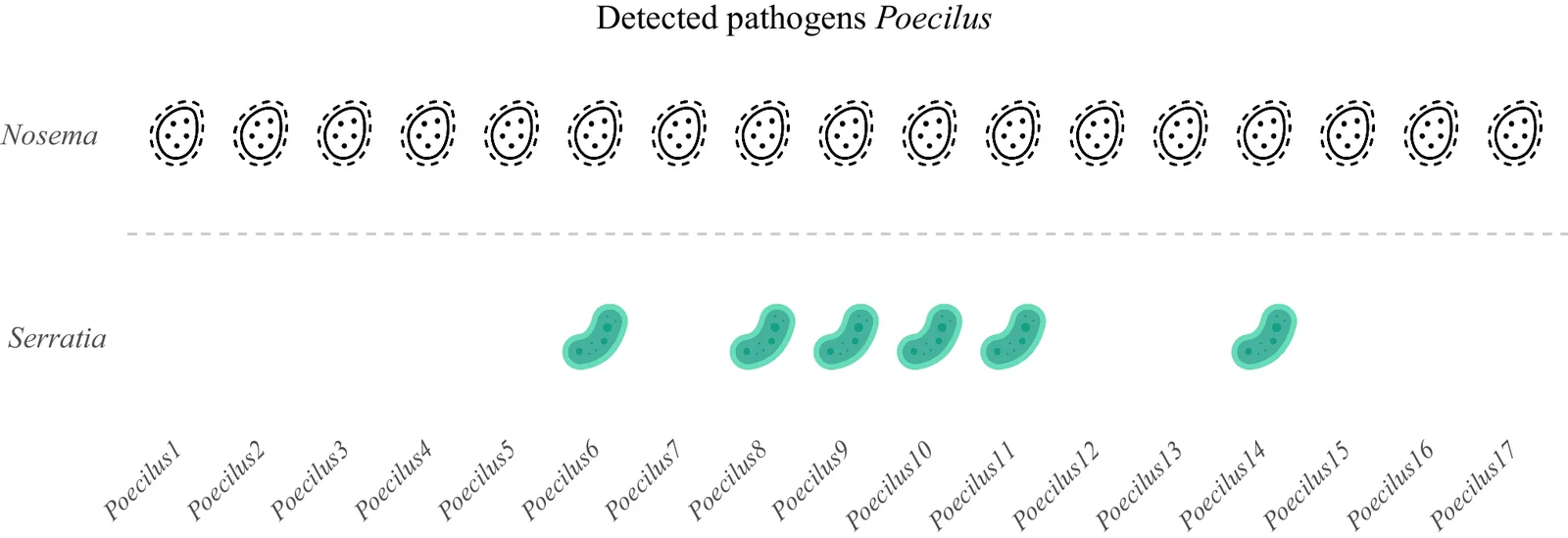
Pathogens presence and absence of *Nosema* and *Serratia *detected by qPCR. Icons were downloaded from Flaticon (https://www.flaticon.com/free-icons/)

Pathogen screening detected *Nosema* in every specimen of both beetle species, while *Serratia* occurred in six *Poecilus* individuals (Fig. 4). Absolute abundance values for all taxa are provided in Table S9.

**Fig. 5:**
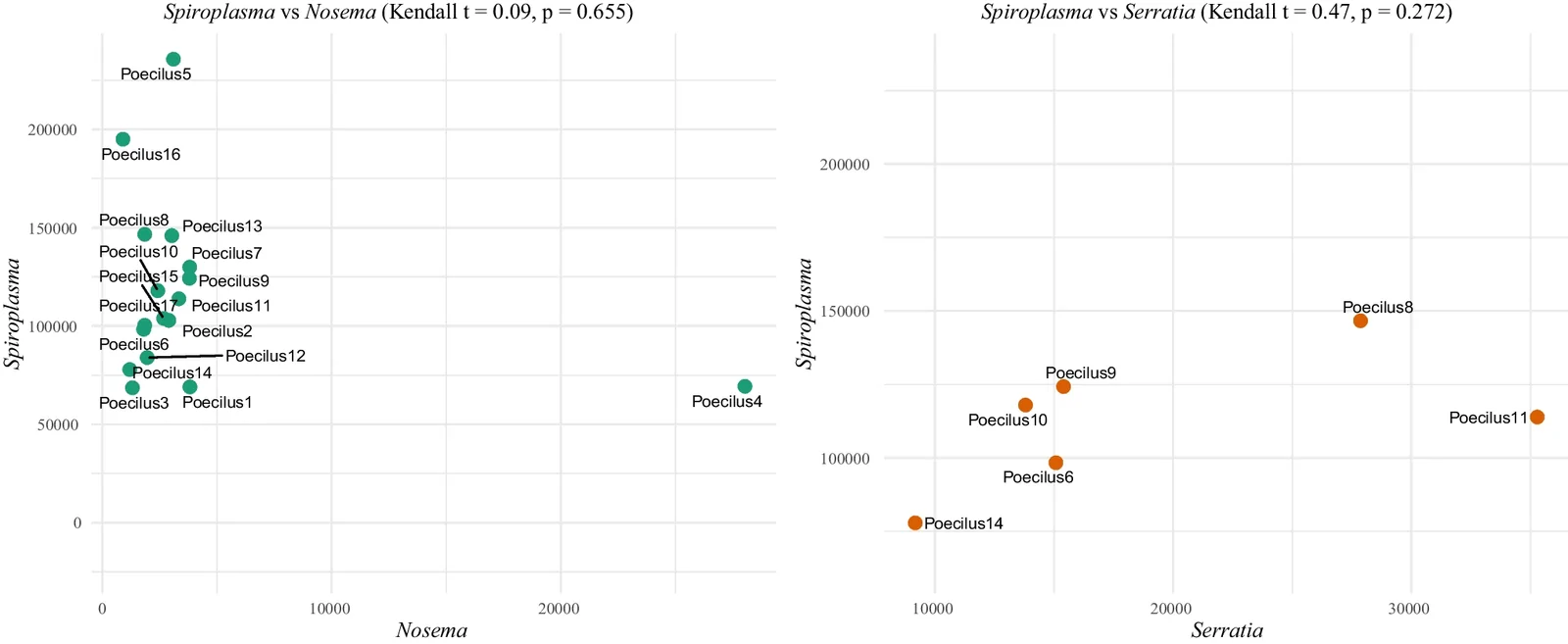
Distribution of Kendall correlation coefficients for *Spiroplasma* versus *Nosema* and *Spiroplasma* versus *Serratia*

Kendall correlation test between *Spiroplasma* versus *Nosema* (*t* = 0.09, *p* = 0.655) and *Spiroplasma* versus *Serratia* (*t* = 0.47, *p* = 0.272) did not reveal significant correlations (Fig. 5). However, linear correlation distribution was suggested between *Spiroplasma* versus *Serratia*.

## Discussion

The abundance of *Poecilus* specimens collected from the organically managed tomato field was significantly higher than from the conventionally managed field. This result aligns with previous studies conducted at the same long-term experimental farm, which reported richer and more abundant soil fauna (including carabids) in organic rotations compared to conventional rotations [20, 36]. The gut microbiota of *Poecilus* individuals was dominated by Pseudomonadota, Bacillota, Actinomycetota and Bacteroidota, taxa commonly observed in the gut ecosystems of other ground beetles and terrestrial arthropods [23, 37]. This consistent core at the phylum level underscores a conserved microbial architecture, potentially shaped by shared ecological roles and dietary niches. Despite this higher-level consistency, our study revealed substantial inter-individual variation at finer taxonomic levels, especially in community diversity and structure. Our results at family level indicate that the *Poecilus* gut harbours both dominant and low-abundance microbial lineages that are conserved across individuals, potentially reflecting co-evolutionary relationships or shared environmental exposure, particularly in organically managed agroecosystems from which most individuals originated. Considering the genus level, core taxa included *Enterobacter*, *Gilliamella*, *Weissella*, *Enterococcus*, *Staphylococcus*, *Carnobacterium*, and *Bacillus*. *Enterobacter*, an unidentified genus within *Enterobacteriaceae*, *Gilliamella*, and *Weissella*, were detected in 100% of samples, further underscoring their ubiquity and potential ecological significance. Finally, at species level, our core microbiota analysis confirms that certain taxa are consistently prevalent (e.g., *Enterobacter hormaechei*, *Enterococcus durans*, *Gilliamella apicola*) suggesting potential adaptation to the *Poecilus* gut niche. These taxa may serve as valuable biological indicators of host health or environmental conditions and could help assess the impact of agricultural practices on insect microbiome composition and, by extension, soil quality [21]. Our analysis was based on OTUs using the traditional 97% similarity cutoff, a widely accepted method for microbial community profiling at the genus-level.

### Microbial Diversity and Environmental Influences

The gut microbiota of *Poecilus* beetles showed marked inter-individual variation in both diversity and composition, reflecting a range of ecological influences such as diet, habitat, and farming practices [22]. The lowest alpha diversity was observed in individuals collected from the CNV farmed field. These individuals’ gut microbiome were overwhelmingly dominated by *Spiroplasma*, with very low evenness and entropy-based metrics, suggesting gut dysbiosis. Although this finding is based on only two CNV specimens (the only beetles captured from that field), it mirrors the *Pseudophonus* results reported by Magagnoli et al. [20] from the same experimental fields. Dysbiosis, defined by reduced microbial diversity in the *Poecilus* gut and the dominance of potentially opportunistic taxa, may be due to environmental stressors [38–40]. One such stressor is agrochemical exposure, as shown by Matsuzaki et al. [41], who demonstrated that exposure to xenobiotics, including pesticides, significantly alters gut microbial composition in animal models. For instance, the impact of land use on the shaping of the gut microbiome composition has been already reported in bumblebees by Fernandez de Landa et al., [42]. While the limited number of specimens collected from CNV farmed sites precludes direct comparisons with those from ORG fields, the CNV-collected *Poecilus* profile, markedly dominated by *Spiroplasma*, is noteworthy and may reflect such agrochemical impacts. This hypothesis remains speculative and requires validation with a larger sample set.

In contrast, individuals exhibiting high Shannon and Simpson values, indicative of more evenly distributed and taxonomically rich communities, are often associated with greater resilience and functional stability in insect gut ecosystems [16, 17]. Finally, the substantial inter-individual variation in alpha diversity metrics observed in our study has been observed in other beetle species. For example, Moldovan et al. [22] found that even in cave-adapted beetles with relatively conserved microbiota at the species level, individual beetles showed distinct abundance patterns, reflecting differences in ecological exposure or physiological state.

### Pathogen Presence and Microbial Interactions

Microbial diversity differences among *Poecilus* individuals prompted investigation into pathogen presence. Notably, this study reports *Nosema ceranae* in Carabidae for the first time. Previously found in *Apis mellifera*, *Aethina tumida*, *Ostrinia nubilalis*, and *Vespa orientalis* [43–46], its detection in *Poecilus* suggests either a broader host range or possible acquisition via scavenging on *Nosema*-infected deceased bees and other insects. Moreover, *Serratia*, an opportunistic insect and human pathogen [47, 48], was found in only six individuals, much less than in *Pseudophonus* from the same sites [20], indicating potential species-specific resistance or microbial suppression mechanisms. *Spiroplasma*, highly abundant in samples collected in CNV, has variable roles across insects, from symbiont to pathogen [18, 19]. Its dominance in P1, with minimal diversity, may indicate dysbiosis, potentially linked to environmental stress. While *Spiroplasma* has been linked to immune benefits and pathogen suppression (e.g., trypanosome resistance in tsetse flies; lower *Nosema* in *Bombus pauloensis*) [49], no significant correlation with *Nosema* (*τ* = 0.09, *p* = 0.655) or *Serratia* (*τ* = 0.47, *p* = 0.272) was found here. Notably, *Spiroplasma*-dominated P1 lacked *Serratia*, possibly due to competitive exclusion or immune factors. Overall, these results suggest a complex, context-dependent role for *Spiroplasma* in insect gut ecology, potentially as a gatekeeper or dysbiosis marker. Broader functional studies will be key to understanding its impact on host health and microbiome stability.

### Predicted Functional Roles and Environmental Origins of Dominant Gut Microbes in Poecilus Beetles

Beyond the potential influence of *Spiroplasma* on pathogen dynamics, we examined the predicted metabolic functions of dominant bacterial genera in the *Poecilus* gut microbiome. Functional profiling highlights how dominant bacterial taxa may contribute to host nutrition, stress resilience, and ecological adaptability, key traits for beetles thriving in dynamic agricultural landscapes. Because these predictions rely on 16S-based KEGG/RAST inferences rather than shotgun metagenomics or transcriptomics, they should be viewed as hypotheses about community‐level potential, not direct evidence of active metabolism. To contextualise these roles, Table S10 and S11 summarises predicted functional scores for core bacterial genera. Table 2 complements this by grouping taxa into three categories based on their predicted ecological roles: (1) core metabolic symbionts involved in digestion and nutrient processing, (2) taxa associated with detoxification and stress tolerance, and (3) supplementary contributors to micronutrient synthesis and gut homeostasis. The structure also reflects probable acquisition routes, such as soil contact, prey ingestion, or interaction with plant surfaces.

**Table 2:** Functional roles and ecological significance of core bacterial genera identified in the gut microbiota of *Poecilus* beetles in our study

Genus	Function summary	Abundance/prevalence in *Poecilus*	Interpretation	Likely route of acquisition	Reference
*Gilliamella*	High in carbohydrate metabolism, fatty acid biosynthesis (5.14), aromatic compound degradation (3.39)	Highly prevalent and abundant (especially in P1 and P2)	Central metabolic hub; likely supports digestion, energy harvesting, and detoxification	Horizontal transfer from pollinators or plant-associated environments	[50, 51]
*Weissella*	High fatty acid biosynthesis (5.07) and nitrogen metabolism (12.16)	Very abundant and prevalent; often dominant	Core symbiont contributing to nutrient storage and nitrogen cycling	Plant material, soil, or prey-associated microbiota	[52–54]
*Enterococcus*	High monosaccharide degradation (22.33), broad carbohydrate metabolism	High prevalence, moderate abundance	Likely supports sugar metabolism and energy generation	Gut of prey organisms, soil, environmental exposure	[55–57]
*Lactobacillus*	Nitrogen metabolism and vitamin synthesis notable	Low-to-moderate abundance, variable prevalence	Likely plays a role in nutritional balance and gut homeostasis	Plant surfaces, prey gut, environmental detritus	[58]
*Apilactobacillus*	High in nitrogen metabolism (16.10), vitamin synthesis (12.71)	Possibly overlapping with Lactobacillus taxa	Supportive symbiont in biosynthetic and metabolic functions	Fermented substrates, plant material, shared origin with *Lactobacillus*	[59]
*Enterobacter*	High in toxin resistance, aromatic compound metabolism (5.36)	Present in many samples, moderate-to-high abundance (P1–3)	May facilitate detoxification and adaptation to environmental stressors	Soil, plants, and insect prey	[60, 61]
*Carnobacterium*	Moderate in amino acid synthesis (280), protein degradation (289), toxic compound resistance (87)	High prevalence, moderate relative abundance	First report in insects; may contribute to detoxification, stress resilience, and possibly chitin breakdown via alpha-chitinase activity	Soil, composted manure, or insect prey	[62, 63]
*Bacillus*	Strong protein degradation (26.78), amino acid synthesis (24.75), antibiotic resistance (7.17)	Present at low levels across most samples	Likely a supportive contributor to protein turnover and stress resilience	Soil, decaying organic matter, prey	[64]
*Staphylococcus*	Highest amino acid synthesis (27.42) and strong protein degradation (22.84). Minor hormone function	Present but low abundance, moderate prevalence	Functionally active, but ecological relevance may be minor; protein degradation may relate to carnivorous diet	Environmental surfaces, prey organisms	[65]
*Lactococcus*	Highest vitamin synthesis (12.46)	Moderate abundance, high prevalence	Micronutrient provider; may help maintain host nutrition	Fermented plant material, soil, gut of prey	[66]

Among core metabolic symbionts, *Gilliamella* and *Weissella* were particularly frequent. *Gilliamella*, abundant in some individuals, showed strong functional potential for carbohydrate metabolism, aromatic compound degradation, and fatty acid biosynthesis. These functions support energy harvesting and pathogen resistance [67]. Notably, *Gilliamella* has been identified as a key degrader of plant-derived polysaccharides in the honey bee gut, particularly cellulose, hemicellulose, and pectin [50, 51]. The species *Gilliamella apicola*, previously described in honey bees and bumble bees [68, 69], was prevalent in *Poecilus*, suggesting horizontal transfer from pollinator-associated environments.

*Weissella*, similarly dominant, showed high predicted potential for nitrogen cycling and fatty acid biosynthesis and is frequently found in agricultural soils [70], raw tomatoes [71], and the slime of garden snails [72]. This implies diverse acquisition routes, including direct soil contact, crop (tomatoes) surface exposure and trophic exposure. Additionally, *Weissella* is recognised for its production of bacteriocins and bioactive metabolites like dextran (which can promote the growth of other beneficial microbes) [52], riboflavin [53], and antioxidants [54].

*Enterococcus* and *Enterobacter* were also prevalent, contributing to sugar metabolism and detoxification functions, respectively. Among the three prevalent *Enterococcus* species detected in *Poecilus*, *E. durans*, and *E. faecium* were identified. Both *E. durans* and *E. faecium* are widespread environmental commensals commonly found in soil, water, plants, and the gastrointestinal tracts of animals and insects [73–75]. In insects, *Enterococcus* species have been shown to modulate host immunity and development [55–57]. Although *Enterococcus mundtii* was not dominant in *Poecilus*, it is noteworthy for its protective role against pathogens, including demonstrated antimicrobial activity and enhanced survival of *Tribolium castaneum* larvae challenged with *Bacillus thuringiensis* [76]. These findings suggest that *Enterococcus* strains in *Poecilus* likely originate from multiple environmental sources and may contribute to host defence and physiological regulation. Among Enterobacteriaceae, *Enterobacter hormaechei* stood out due to its roles in promoting growth, stabilising the microbiota, and inhibiting pathogens in insects such as housefly larvae [60]. Moreover, this bacterium has demonstrated resilience to copper toxicity and supports host motility under Cu^2^⁺ stress [61]. This is especially relevant as most *Poecilus* were sampled from organic farms where copper sulphate is widely used as a fungicide [77]. Thus, the presence of *E. hormaechei* in the *Poecilus* gut may reflect an adaptive microbial response to copper exposure in the beetles’ environment, potentially supporting detoxification processes and enhancing ecological fitness.

*Carnobacterium*, reported here for the first time in an insect host, adds a novel component to insect gut microbiota. Detected at moderate abundance, it showed high predicted functional activity (e.g., amino acid synthesis, protein degradation, toxin resistance), suggesting roles in nutrient processing and stress resilience. Some strains also produce chitinases, potentially aiding digestion of insect prey [62, 63]. Although not previously confirmed in terrestrial insects [78], *Carnobacterium* is known from meat, fish, and manure [79–81], indicating *Poecilus* may acquire it via compost-amended soils, prey, or decomposing material.

Together, the gut microbial community of *Poecilus* appears to hypothetically support a broad array of physiological functions, from energy metabolism and detoxification to immune modulation and stress tolerance. These results align with findings in other insects [24, 37] and reinforce the importance of the gut microbiome in supporting beetle health and ecological function across agroecosystems. This broad functional repertoire is substantiated by our enzymatic function predictions (Figure S9), which illustrate that enzymes involved in amino acid metabolism and protein degradation are consistently among the most abundant and widely distributed functional categories across individual beetle microbiomes. Such core functions are highly conserved, suggesting their central role in supporting host nutrition and resilience. At the same time, the relative predicted activity for these functions exhibits notable inter-individual variation, with certain samples displaying lower functional scores that correspond to reduced microbial diversity. This functional convergence underscores the ecological importance of a metabolically versatile microbiome in facilitating adaptation to protein-rich or variable diets and providing resilience to environmental change.

### Beta Diversity Outperforms Alpha Diversity in Predicting Microbial Function

Multiple linear regressions showed that alpha diversity indices (Shannon, Simpson, Richness, Chao1, Zahl) did not significantly predict functional potential across 12 pathways (all *p* > 0.05). This suggests that alpha diversity alone does not reflect redundancy or dominance within the microbiome and that high richness (e.g., in P4, P12) may involve rare taxa with limited metabolic roles [82, 83].

In contrast, PCoA (Bray–Curtis) revealed three compositional clusters. Although PERMANOVA found no significant separation, these PCoA-derived clusters differed markedly in their predicted functional profiles: amino-acid biosynthesis (*p* < 0.01), protein degradation (*p* < 0.005), and antibiotic-resistance pathways (*p* < 0.005) all varied significantly among clusters (Table S10). These findings suggest that beta diversity (community composition) is more informative than alpha diversity for predicting functional traits, linking microbiome structure to potential physiological roles or environmental exposure.

### Study Limitations

This study has some limitations that should be considered. No extraction blanks were included during DNA sequencing, which prevents us from fully excluding potential low-level contamination, although no typical reagent-derived taxa were detected. In addition, only two gut samples came from conventionally (CNV) managed fields, limiting statistical power. Consequently, any findings involving the CNV group should be viewed as preliminary, even though the observed trends are consistent and biologically plausible.

### Challenges of Studying the Gut Microbiome of Understudied Organisms

While our combined NGS and qPCR approach provides robust insights into the *Poecilus* gut microbiota, we recognise several methodological limitations. Notably, NGS-based amplicon sequencing may overestimate or underestimate certain microbial groups due to primer bias, amplification efficiency, and variable 16S rRNA gene copy numbers. Conversely, our qPCR quantification could be affected if primers do not efficiently anneal to the specific bacterial strains present in insects, potentially leading to underestimation of true abundances. This is a common challenge, as most available qPCR primers are designed for bacteria for specific animal models (e.g., mammalian or honey bee microbes). Our findings therefore highlight the need for future work to optimise and validate primer sets specifically tailored to the gut microbiota of understudied organisms, in order to improve quantitative accuracy in both NGS and qPCR studies.

## Conclusions

Our study reveals that the *Poecilus* gut microbiome ranges from low-diversity, potentially dysbiotic communities to rich, functionally diverse assemblages, shaped by both host and environmental factors. Core functions like amino acid metabolism and protein degradation remain conserved, highlighting their ecological relevance.

Despite the limited representation from conventional farming systems in our sampling, our results provide important insights into how agricultural management shapes the gut microbiome of *Poecilus*. Future studies with more balanced sampling designs are essential to fully capture the spectrum of microbiome responses to different farming practices and to identify microbial markers that may predict soil and ecosystem health.

Overall, *Poecilus* emerges as a promising bioindicator for monitoring environmental change, supporting the integration of microbial metrics into sustainable agricultural strategies.

Supplementary Information.

## Supplementary Information

Below is the link to the electronic supplementary material.

ESM 1 PDF (1.38 MB)

## Data Availability

NGS 16S rRNA sequence data have been deposited at NCBI repository Sequence Read Archive (SRA) and are publicly available as of the date of publication. Accession numbers are listed in the key resources table. Bio-project n° PRJNA798890, Bio-sample numbers SAMN25144083 - SAMN25144087 - SAMN25144088 - SAMN25144092 - SAMN25144097 - SAMN25144098 - SAMN25144102 - SAMN25144107 - SAMN25144108 - SAMN25144109 - SAMN25144110 - SAMN25144111 - SAMN25144117 - SAMN25144123 - SAMN25144126 - SAMN25144133 - SAMN25144134. The naming convention for the samples whose sequences have been uploaded to the NCBI repository is provided in Table S12.
