# Supplementary material for: The Ground Beetle Poecilus (Carabidae) Gut Microbiome and Its Functionality
Source: Microb Ecol. 2025 Jul 30;88(1):83. doi: 10.1007/s00248-025-02579-0 (PMC12310908; doi:10.1007/s00248-025-02579-0)
Supplement: Supplementary file 1 — PDF (1.38 MB) [file 248_2025_2579_MOESM1_ESM.pdf]

**Supplementary Materials for:**

# The Ground Beetle *Poecilus* (Carabidae) Gut Microbiome and Its Functionality

**Authors:** Chiara Braglia[1†], Simone Cutajar[1,2†], Serena Magagnoli[1], Diana Asciano[1], Giovanni Burgio[1], Diana Di Gioia[1], Loredana Baffoni[1], Daniele Alberoni[1*].

**Affiliations:**

[1] Dipartimento di Scienze e Tecnologie Agro-Alimentari (DISTAL), Università di Bologna, Viale Fanin 42, 40127, Bologna, Italy;

[2] Institute of Earth Systems, L-Università tà Malta, Msida, MSD 2080, Malta.

**Supplementary Table S1.** qPCR-specific primers used in this study.

| | Primer Name | Sequence (5′-3′) | Amplicon size (bp) | Annealing Temperature °C | Melting °C ($T_m$) | Reference |
|---|---|---|---|---|---|---|
| ***Bartonella*** | Bart-Fw | GTGGGAATCTACCTATTTCTACG | 103 | 60 | 75.1 | Kešnerová et al., 2017 |
| | Bart-Rev | AACGCGGGCTCATCTATCTC | | | | |
| ***Bifidobacterium*** | Bif-Fw | TCGCGTCYGGTGTGAAAG | 243 | 60 | 86.8 | Rinttilä et al., 2004 |
| | Bif-Rev | CCACATCCAGCRTCCAC | | | | |
| ***Bombi-lactobacillus*** | Firm4-Fw | AGTCGAGCGCGGGAAGTCA | 169 | 59 | 85.5 | Kešnerová et al., 2017 |
| | Firm4-Rev | AGCCGTCTTTCAACCAGCACT | | | | |
| **Eubacteria** | Eub338-Fw | ACTCCTACGGGAGGCAGCAG | 200 | 60 | - | Lane, 1991 |
| | Eub518-Rev | ATTACCGCGGCTGCTGG | | | | |
| ***Lactobacillus*** | Firm5-Fw | GCAACCTGCCCTWTAGCTTG | 118 | 59 | 81.5 | Kešnerová et al., 2017 |
| | Firm5-Rev | GCCCATCCTKTAGTGACAGC | | | | |
| ***Gilliamella*** | Gill-Fw | CTTTGTTGCCATCGGTTAGGCC | 160 | 56 | 84.5 | Kešnerová et al., 2017 |
| | Gill-Rev | CCGCTTGCTCTCGCGAGG | | | | |
| ***Snodgrassella*** | Snod-Fw | CTTAGAGATAGGAGAGTGCCTT | 132 | 58 | 85.3 | Kešnerová et al., 2017 |
| | Snod-Rev | AACTTAATGATGGCAACTAATGACAA | | | | |
| ***Spiroplasma*** | BS1 | AAGTCGAACGGGGTGCTT | 976 | 57 | 76.7 | Meeus et al., 2012 |
| | BS2 | TGCACCACCTGTCTCAATGT | | | | |
| ***Serratia*** | luxS1-Fw | TGCCTGGAAAGCGGCGATGG | <300 | 61 | 86.4 | Joyner et al., 2014 |
| | LuxS2-Rv | CGCCAGCTCGTCGTTGTGGT | | | | |
| ***Nosema ceranae*** | Nc841f | GAGAGAACGGTTTTTTGTTTGAGA | 140 | 60 | 77.3 | Huang and Solter, 2013 |
| | Nc980r | ATCCTTTCCTTCCTACACTGATTG | | | | |
| ***Enterobacter*** | ECST748F | AGAAATTCCAAACGAACTTG | 92 | 60 | 78.3 | Ludwig et al., 2004 |
| | ENC854R | CAGTGCTCTACCTCCATCATT | | | | |
| ***Illumina adapter—V3-V4 Region of 16S rRNA gene*** | Pro341-F | *AATGATACGGCGACCACCGAGATCTACACTCTTTCCCTACACGACGCTCTTCCGATCTCCTACGGGAGGCAGCAG*-**CCTACGGGNGCASCAG** | 460 | 55 | - | Takahashi et al. 2014 |
| | Pro805-R | *CAAGCAGAAGACGGCATACGAGATNNNNNNGTGACTGGAGTTCAGACGTGTGCTCTTCCGATCT*-**GACTACNVGGGTATCTAATC**C | | | | |

**Supplementary Table S2.** Specific pathways considered in the functional analysis and the relative class groups.

| Metabolic pathway class | Specific class pathways |
|---|---|
| **Polysaccharide's degradation** | ▪ cellulase<br>▪ xylanase<br>▪ glucanase<br>▪ polysaccharide |
| **Monosaccharides** | ▪ glucose<br>▪ fructose<br>▪ mannose<br>▪ galactose<br>▪ monosaccharide<br>▪ sugar |
| **Vitamins** | ▪ vitamin<br>▪ biotin<br>▪ thiamin<br>▪ riboflavin<br>▪ cobalamin<br>▪ folate |
| **Nitrogen Metabolism** | ▪ nitrate<br>▪ nitrite<br>▪ nitrogenase<br>▪ ammonia<br>▪ glutamine<br>▪ GlnK |
| **Protein degradation** | ▪ protease<br>▪ peptidase<br>▪ protein degradation<br>▪ deformylase |
| **Alkaloids degradation** | ▪ alkaloid<br>▪ nicotine<br>▪ indole<br>▪ tropane |
| **Hormones** | ▪ hormone<br>▪ auxin<br>▪ cytokinin<br>▪ gibberellin |
| **Metabolism of Aromatic Compounds** | ▪ aromatic<br>▪ phenol<br>▪ benzoate<br>▪ catechol<br>▪ protocatechuate<br>▪ salicylate<br>▪ 4-hydroxybenzoate |
| **Amino Acids synthesis** | ▪ amino acid biosynthesis<br>▪ biosynthesis of amino acids<br>▪ aspartate<br>▪ glutamate<br>▪ methionine<br>▪ tryptophan<br>▪ lysine<br>▪ histidine<br>▪ valine<br>▪ leucine<br>▪ isoleucine |
| **Resistance to antibiotics** | ▪ antibiotic<br>▪ multidrug<br>▪ beta-lactamase<br>▪ tetracycline<br>▪ macrolide<br>▪ vancomycin |
| **Toxic compounds resistance** | ▪ toxic<br>▪ arsenic<br>▪ mercury<br>▪ cadmium<br>▪ copper<br>▪ zinc efflux<br>▪ heavy metal |
| **Fatty Acid Biosynthesis** | ▪ fatty acid biosynthesis<br>▪ acetyl-CoA carboxylase<br>▪ acyl-carrier<br>▪ fab[A-Z] |

**Supplementary Table S3.** Alpha diversity metrics (Shannon, Simpson, richness, Zahl's index and Chao1 richness) across individual *Poecilus* beetles.

| Sample | Shannon | Simpson | Zahl (Shannon entropy) | Richness | Chao1 Richness |
|---|---|---|---|---|---|
| Poecilus 1 | 0.7343006 | 0.5046380 | 0.007691072 | 79 | 91.15 |
| Poecilus 2 | 1.6595669 | 0.6893572 | 1.767608421 | 109 | 131.25 |
| Poecilus 3 | 1.2717321 | 0.6404093 | 1.378541389 | 95 | 139.15 |
| Poecilus 4 | 1.6870988 | 0.6913560 | 1.777854256 | 184 | 291.96 |
| Poecilus 5 | 1.6772153 | 0.6907794 | 1.710429343 | 173 | 289.30 |
| Poecilus 6 | 1.3347564 | 0.6611136 | 1.575804072 | 53 | 107.20 |
| Poecilus 7 | 1.6010245 | 0.6950137 | 1.493601036 | 63 | 91.54 |
| Poecilus 8 | 1.5503853 | 0.6850268 | 1.879215042 | 57 | 66.11 |
| Poecilus 9 | 1.1979060 | 0.6084242 | 0.983420012 | 49 | 56.25 |
| Poecilus 10 | 1.5070874 | 0.6695597 | 1.514355074 | 53 | 63.00 |
| Poecilus 11 | 1.1095500 | 0.5838409 | 0.724575987 | 92 | 137.86 |
| Poecilus 12 | 1.8168078 | 0.6991047 | 1.622895448 | 174 | 222.45 |
| Poecilus 13 | 1.5703777 | 0.6737625 | 1.556027140 | 69 | 75.00 |
| Poecilus 14 | 1.5633207 | 0.6349583 | 1.322478649 | 182 | 240.03 |
| Poecilus 15 | 1.6920631 | 0.6881611 | 2.367435093 | 75 | 125.00 |
| Poecilus 16 | 1.2960868 | 0.6142737 | 1.295129677 | 69 | 89.08 |
| Poecilus 17 | 1.8894880 | 0.7110901 | 2.036127165 | 126 | 178.71 |

**Supplementary Table S4.** Phylum-level core microbiota composition of *Poecilus* gut based on prevalence and mean relative abundance (%).

| Phylum | Prevalence | Relative Mean Abundance (%) |
|---|---|---|
| *Pseudomonadota* | 1.000 | 52.300 |
| *Bacillota* | 1.000 | 23.300 |
| *Actinomycetota* | 1.000 | 8.620 |
| *Mycoplasmatota* | 0.647 | 8.330 |
| *Bacteroidota* | 1.000 | 6.740 |
| *Verrucomicrobiota* | 0.765 | 0.617 |
| *Spirochaetota* | 0.412 | 0.074 |
| *Cyanobacteriota* | 0.588 | 0.017 |
| *Chloroflexota* | 0.706 | 0.016 |
| *Planctomycetota* | 0.235 | 0.004 |
| *Saccharimonadota* | 0.235 | 0.004 |
| *Tectimicrobiota* | 0.294 | 0.002 |
| *Gemmatimonadota* | 0.118 | 0.001 |
| *Nitrospirota* | 0.118 | 0.001 |

**Supplementary Table S5.** Family-level composition of core gut microbiota in *Poecilus* beetles, showing prevalence and relative abundance (%) across individuals.

| Family | Prevalence | Relative Mean Abundance (%) |
|---|---|---|
| Others | 1.000 | 44.70 |
| Orbaceae | 1.000 | 30.40 |
| Leuconostocaceae | 0.941 | 13.40 |
| Streptococcaceae | 0.824 | 4.330 |
| Enterobacteriaceae | 1.000 | 3.020 |
| Enterococcaceae | 0.941 | 2.020 |
| Staphylococcaceae | 0.882 | 1.450 |
| Carnobacteriaceae | 0.941 | 0.356 |
| Lactobacillaceae | 0.941 | 0.294 |
| Bacillaceae | 0.882 | 0.039 |

**Supplementary Table S6.** Prevalence and percentage relative abundance of bacterial Operational Taxonomic Units (OTUs) associated with *Poecilus* beetles.

| **OTU** | **Prevalence** | **Relative Mean Abundance (%)** |
|---|---|---|
| *Bacillus* sp. | 0.882 | 0.040 |
| *Carnobacterium* sp. | 0.941 | 0.356 |
| *Enterobacter hormaechei* | 0.824 | 0.201 |
| *Enterobacter* sp.1 | 0.882 | 0.092 |
| *Enterobacter* sp.2 | 0.882 | 0.076 |
| *Enterobacter* sp.3 | 0.941 | 0.719 |
| *Enterobacteriaceae* | 0.941 | 0.773 |
| *Enterococcus durans* | 1.000 | 0.333 |
| *Enterococcus faecium* | 0.941 | 0.057 |
| *Enterococcus* sp. | 0.941 | 29.000 |
| *Gilliamella apicola* | 0.941 | 0.294 |
| *Gilliamella* sp.1 | 0.824 | 4.320 |
| *Gilliamella* sp.2 | 0.941 | 0.455 |
| *Lactobacillus* sp. | 1.000 | 2.390 |
| *Lactococcus* sp. | 1.000 | 1.280 |
| *Staphylococcus* sp. | 0.882 | 1.450 |
| *Weissella* sp. | 0.941 | 13.400 |
| Other | 1.000 | 44.700 |

**Supplementary Table S7.** Prevalence and percentage relative abundance of bacterial Genera associated with *Poecilus* beetles.

| Genus | Prevalence | Relative Mean Abundance (%) |
|---|---|---|
| *Bacillus* | 0.882 | 0.039 |
| *Carnobacterium* | 0.941 | 0.356 |
| *Enterobacter* | 0.941 | 0.293 |
| Enterobacteriaceae | 1.000 | 2.39 |
| *Enterococcus* | 0.941 | 2.02 |
| *Escherichia-Shigella* | 1.000 | 0.333 |
| *Gilliamella* | 1.000 | 30.40 |
| *Lactobacillus* | 0.941 | 0.294 |
| *Lactococcus* | 0.824 | 4.33 |
| Others | 1.000 | 44.70 |
| *Staphylococcus* | 0.882 | 1.45 |
| *Weissella* | 0.941 | 13.40 |

**Supplementary Table S8.** Number of functional pathways of each core gut microbiome taxa from genome analysis on KEGG and RAST SEED of the selected classes.

| | ***Amino Acids synthesis*** | ***Fatty Acid Biosynthesis*** | ***Monosaccharides*** | ***Nitrogen Metabolism*** | ***Protein degradation*** | ***Resistance to antibiotics*** | ***Toxic compounds resistance*** | ***Vitamins*** | ***Alkaloids degradation*** | ***Hormones*** | ***Metabolism of Aromatic Compounds*** | ***Polysaccharides degradation*** |
|---|---|---|---|---|---|---|---|---|---|---|---|---|
| ***Apilactobacillus*** | 27 | 4 | 10 | 19 | 37 | 3 | 3 | 15 | 0 | 0 | 0 | 0 |
| ***Bacillus*** | 535 | 55 | 234 | 174 | 579 | 155 | 75 | 177 | 6 | 2 | 86 | 84 |
| ***Carnobacterium*** | 280 | 44 | 211 | 143 | 289 | 59 | 87 | 118 | 4 | 5 | 16 | 11 |
| ***Enterobacter*** | 783 | 123 | 562 | 379 | 663 | 233 | 193 | 235 | 12 | 3 | 189 | 150 |
| ***Enterococcus*** | 370 | 61 | 407 | 185 | 416 | 77 | 77 | 186 | 2 | 2 | 18 | 22 |
| ***Gilliamella*** | 217 | 47 | 136 | 102 | 184 | 41 | 21 | 81 | 4 | 4 | 31 | 46 |
| ***Lactobacillus*** | 142 | 15 | 169 | 97 | 225 | 33 | 42 | 83 | 0 | 1 | 8 | 8 |
| ***Lactococcus*** | 183 | 44 | 141 | 98 | 187 | 43 | 37 | 111 | 3 | 3 | 17 | 24 |
| ***Staphylococcus*** | 317 | 35 | 124 | 103 | 264 | 59 | 50 | 129 | 6 | 7 | 30 | 32 |
| ***Weissella*** | 218 | 50 | 150 | 120 | 215 | 49 | 23 | 138 | 0 | 0 | 12 | 12 |

**Supplementary Table S9.** Absolute abundance of targeted microorganisms detected in qPCR. Data are expressed in 16S rRNA copies/gut, spores/gut for *Nosema*, and *luxS* gene copies/gut for *Serratia.*

| Specie | Total Bacteria | *Bartonella* | *Bifidobacterium* | *Bombilactobacillus* | *Enterobacter* | *Snodgrassella* | *Gilliamella* | *Spiroplasma* | *Nosema* | *Serratia* |
|---|---|---|---|---|---|---|---|---|---|---|
| **Poecilus1** | 3818515.43 | 24813.08 | | | | | | 69015.90 | 3819.43 | |
| **Poecilus2** | 235403.7094 | | | | | | | 102865.8325 | | |
| **Poecilus3** | 102581868.75 | 71762.30 | | | 2003186.67 | | 278178.74 | 68570.07 | 1312.47 | |
| **Poecilus4** | 44804901.56 | | 16278.62 | 6737.53 | 465685.78 | | 442820.84 | 69309.72 | 28022.38 | |
| **Poecilus5** | 47888437.50 | | 19730.19 | 4330.47 | 429063.11 | | 269122.34 | 235674.35 | 3101.78 | |
| **Poecilus6** | 44659148.44 | 9453.79 | | | 170025.33 | | 65445.68 | 98321.26 | 1802.41 | 15078.39 |
| **Poecilus7** | 14429264.06 | | | | 159647.56 | | 26996.02 | 129927.91 | 3808.90 | |
| **Poecilus8** | 13250803.13 | 38212.98 | 3470.12 | 6331.22 | 129980.00 | | 2295932.67 | 146637.65 | 1848.43 | 27870.50 |
| **Poecilus9** | 16107677.34 | | | 4169.32 | 38536.00 | | 598811.83 | 124238.92 | 3795.50 | 15401.81 |
| **Poecilus10** | 246181.86 | 16371.81 | | 4340.45 | | | 50399.30 | 117947.98 | 2415.76 | 13802.58 |
| **Poecilus11** | 59381775.00 | 13154.36 | 2066091.13 | 3839.07 | 24996.44 | 641.54 | 678676.39 | 113868.13 | 3339.10 | 35290.72 |
| **Poecilus12** | 27328567.97 | 37463.65 | 785570.01 | 6178.51 | 155122.89 | 1232.99 | 1406265.53 | 83921.12 | 1955.22 | |
| **Poecilus13** | 2079083.06 | | | 4750.10 | 48203.33 | | 176320.51 | 145946.87 | 3030.75 | |
| **Poecilus14** | 48461451.56 | 15076.36 | | 4604.21 | 1021083.11 | | 2160530.42 | 77834.66 | 1194.90 | 9174.16 |
| **Poecilus15** | 987094031.25 | | | | 602909.33 | | 121649081.25 | 103848.51 | 2670.95 | |
| **Poecilus16** | 26061967.97 | | | 1842.47 | 610879.33 | | 2084363.53 | 195002.31 | 899.43 | |
| **Poecilus17** | 42970195.31 | | | 3848.15 | 1861236.44 | | 418984.06 | 100370.06 | 1846.85 | |

**Supplementary Table S10.** Summary of linear regression models testing the association between predicted functional classes and microbial diversity metrics (Shannon, Simpson, and Richness) across *Poecilus* gut samples. The table reports the $R^2$, adjusted $R^2$, model p-values, and whether any predictor was statistically significant for each predicted functionality. No significant associations were observed across models.

| **Functionality** | **$R^2$** | **Adjusted $R^2$** | **Model *p*-value** | **Significant predictors** |
|---|---|---|---|---|
| Amino Acids synthesis | 0.308 | -0.007 | 0.4726 | No |
| Fatty Acid Biosynthesis | 0.444 | 0.192 | 0.2018 | No |
| Mono saccharides | 0.233 | -0.116 | 0.6557 | No |
| Nitrogen Metabolism | 0.465 | 0.221 | 0.1724 | No |
| Protein degradation | 0.166 | -0.213 | 0.8128 | No |
| Resistance to antibiotics | 0.166 | -0.213 | 0.8135 | No |
| Toxic compounds resistance | 0.344 | 0.046 | 0.389 | No |
| Vitamins | 0.297 | -0.022 | 0.4975 | No |
| Alkaloids degradation | 0.374 | 0.089 | 0.327 | No |
| Hormones | 0.377 | 0.093 | 0.3215 | No |
| Metabolism of Aromatic Compounds | 0.258 | -0.079 | 0.5927 | No |
| Polysaccharides degradation | 0.074 | -0.346 | 0.9658 | No |

**Supplementary Table S11.** Statistical association between predicted microbial functionality and beta diversity clustering (PCoA Bray–Curtis).

| **Functionality** | **LM *p*- value** | **Kruskal *p*- value** |
|---|---|---|
| Amino Acids synthesis | 0.001265325 | 0.01022077 |
| Fatty Acid Biosynthesis | 0.1025224 | 0.055549754 |
| Monosaccharides | 0.2790236 | 0.612334737 |
| Nitrogen Metabolism | 0.4402584 | 0.227963117 |
| Protein degradation | 2.55E-05 | 0.003298506 |
| Resistance to antibiotics | 4.65E-05 | 0.003298506 |
| Toxic compounds resistance | 0.02114157 | 0.047471818 |
| Vitamins | 0.001400122 | 0.011931523 |
| Alkaloids degradation | 0.001388224 | 0.011931523 |
| Hormones | 0.001114017 | 0.011931523 |
| Metabolism of Aromatic Compounds | 0.001214738 | 0.011931523 |
| Polysaccharides degradation | 8.77E-06 | 0.005569354 |

**Supplementary Table S12.** Name correspondence when differently cited in the database (eg. NCBI) and supplementary figures.

| ID | Short name | Abbreviated name (Sample name at field collection) | NCBI RefSeq (Run Browser – SRA) |
|---|---|---|---|
| Poecilus1 | P1 | A (iA) | SRR17688595 - SRX13851879 |
| Poecilus2 | P2 | G (iG) | SRR17688587 - SRX13851887 |
| Poecilus3 | P3 | Q7 (i7) | SRR17688625 - SRX13851849 |
| Poecilus4 | P4 | Q11 (i11) | SRR17688620 - SRX13851854 |
| Poecilus5 | P5 | Q12 (i12) | SRR17688619 - SRX13851855 |
| Poecilus6 | P6 | Q17 (i17) | SRR17688615 - SRX13851859 |
| Poecilus7 | P7 | Q22 (i22) | SRR17688609 - SRX13851865 |
| Poecilus8 | P8 | Q25 (25q) | SRR17688581 - SRX13851893 |
| Poecilus9 | P9 | Q28 (28q) | SRR17688578 - SRX13851896 |
| Poecilus10 | P10 | Q31 (i31) | SRR17688608 - SRX13851866 |
| Poecilus11 | P11 | Q38 (i38) | SRR17688604 - SRX13851870 |
| Poecilus12 | P12 | Q40 (40q) | SRR17688628 - SRX13851846 |
| Poecilus13 | P13 | Q43 (43q) | SRR17688627 - SRX13851847 |
| Poecilus14 | P14 | Q47 (i47) | SRR17688598 - SRX13851876 |
| Poecilus15 | P15 | Q48 (i48) | SRR17688597 - SRX13851877 |
| Poecilus16 | P16 | Q49 (i49) | SRR17688596 - SRX13851878 |
| Poecilus17 | P17 | Q50 (i50) | SRR17688594 - SRX13851880 |

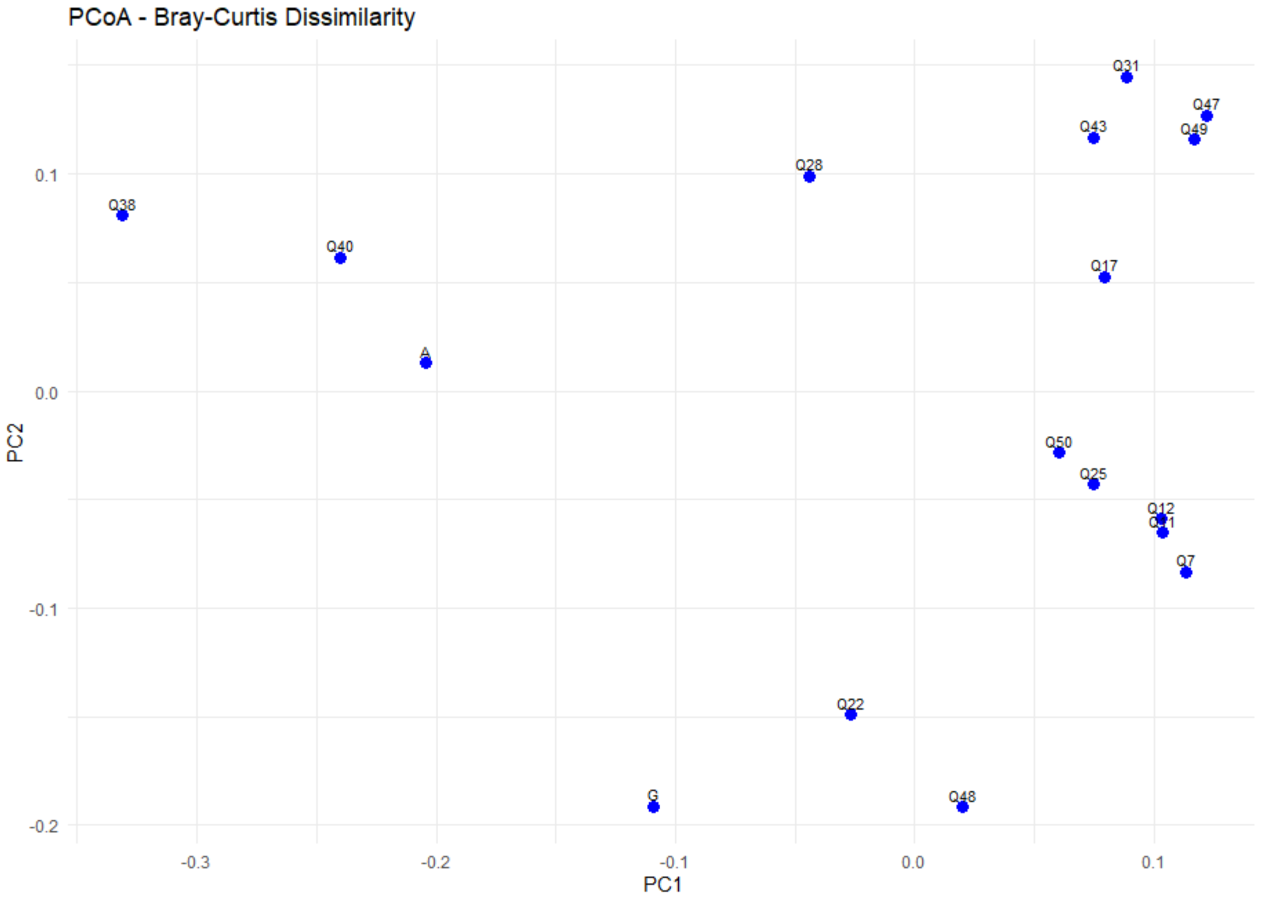


**Supplementary Figure S1.** Principal Coordinates Analysis (PCoA) plot based on Bray-Curtis dissimilarity matrix illustrating beta diversity of gut microbial communities across *Poecilus* samples. Each point represents an individual sample, and spatial separation reflects differences in community composition. No clear clustering by sample group or outlier pattern is observed, indicating considerable inter-individual variation.

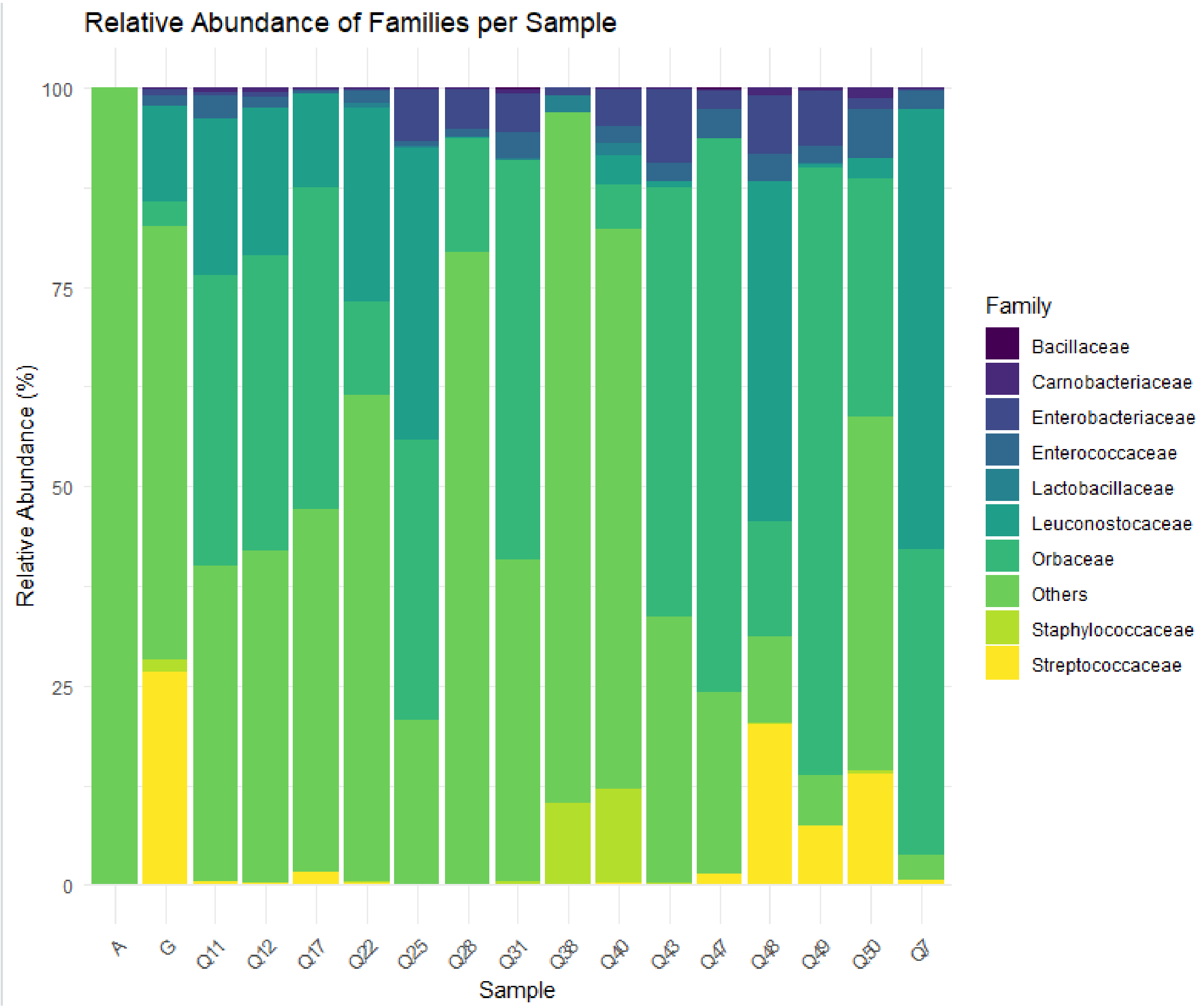


**Supplementary Figure S2**. **Stacked bar plot showing the relative abundance of bacterial families across individual *Poecilus* samples.** Each bar represents the proportional contribution of bacterial families within a sample, revealing inter-individual variation in gut microbiota composition. Prominent families include Orbaceae, Leuconostocaceae, Enterobacteriaceae, and Streptococcaceae, with certain samples dominated by specific families, suggesting potential functional or environmental influences on gut community structure.

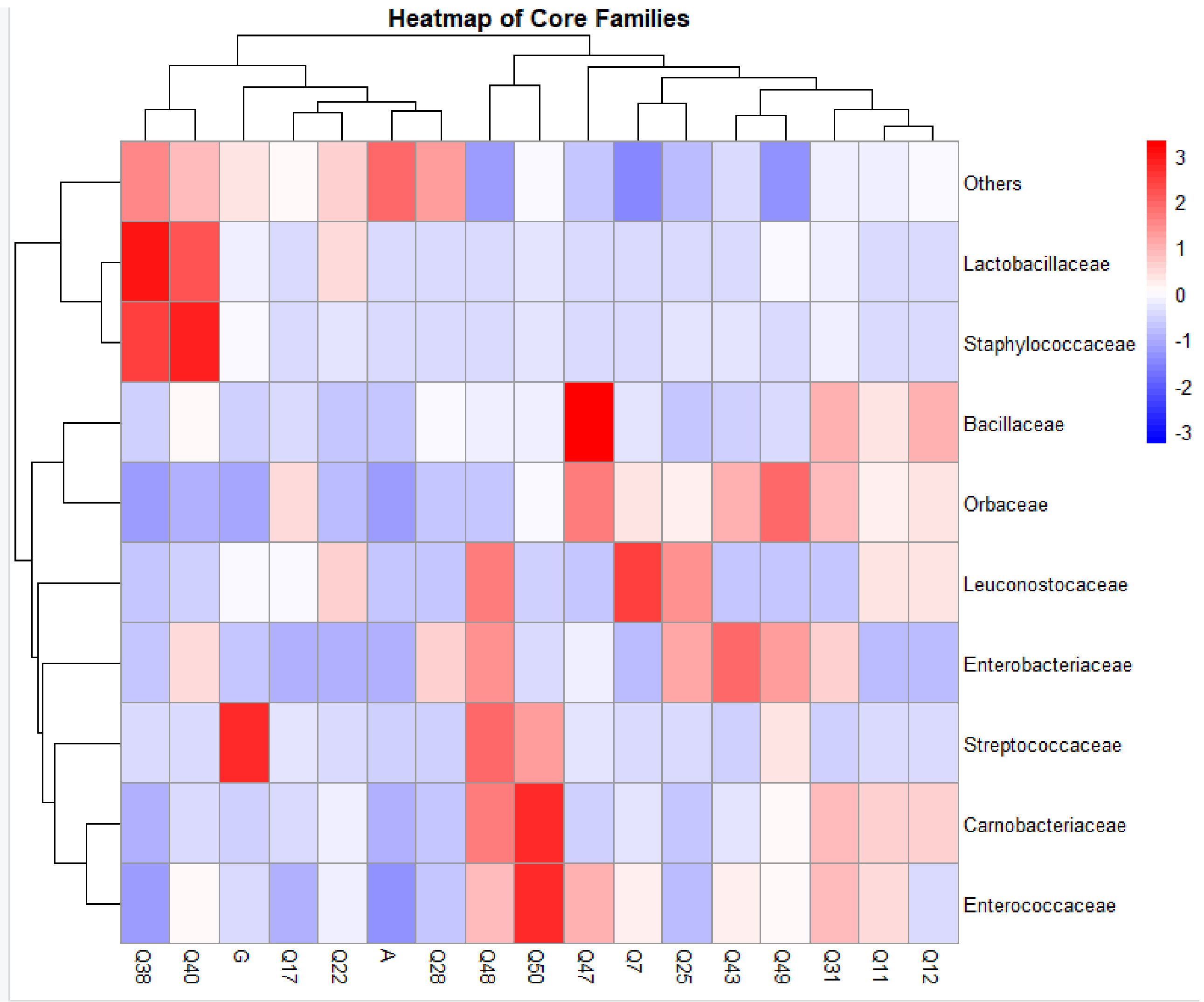


**Supplementary Figure S3**. **Heatmap of core bacterial families across *Poecilus* samples.** The heatmap displays the standardized relative abundance (z-score) of core families across individuals, with clustering of both samples and taxa based on similarity. Warmer tones (red) indicate higher relative abundance, while cooler tones (blue) indicate lower abundance. The clustering highlights distinct patterns in family-level gut microbiota composition, suggesting individual variability.

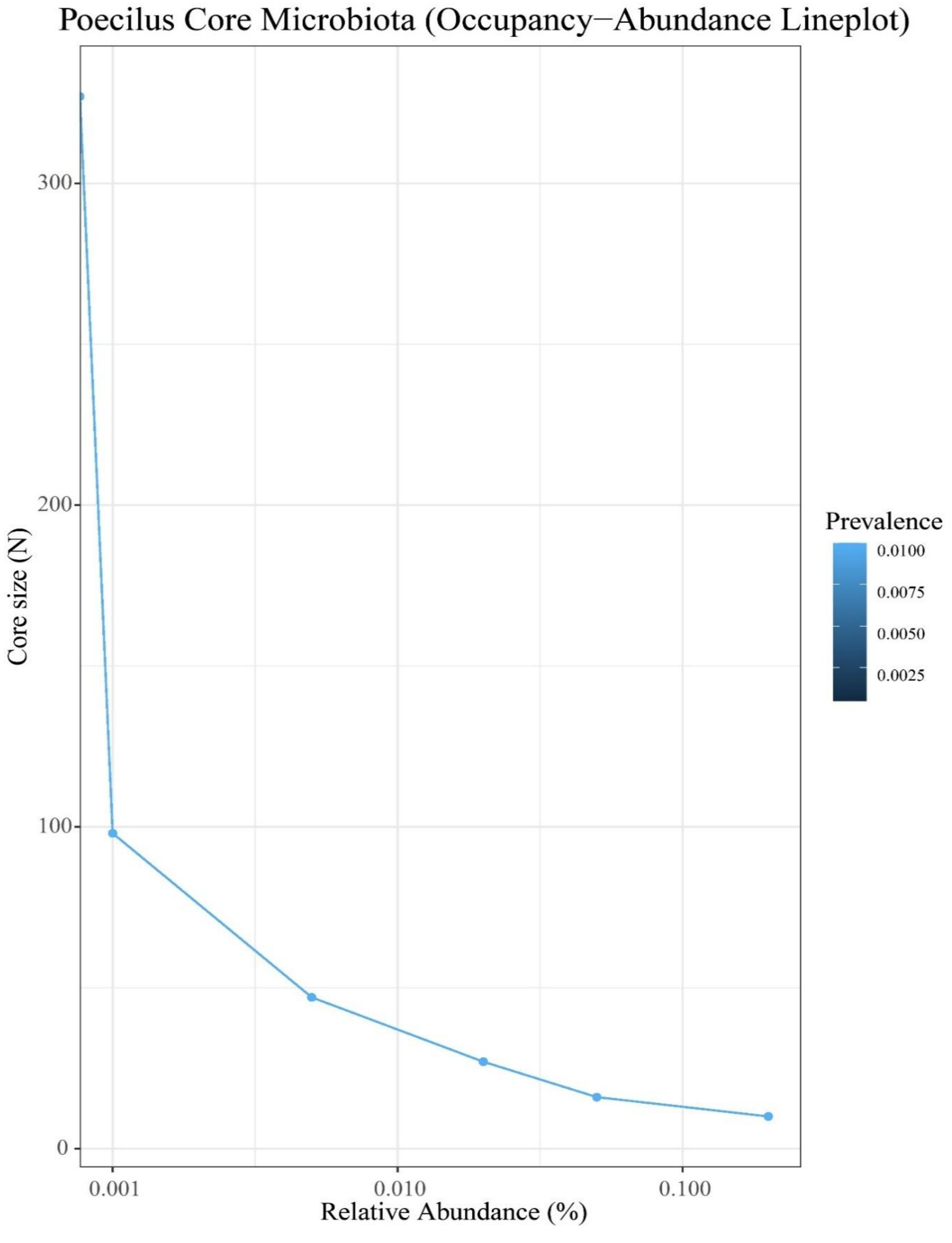


**Supplementary Figure S4**. **Occupancy–abundance relationship of core gut microbiota in *Poecilus* beetles.** Line plot showing the number of core taxa (Y-axis) retained at increasing relative abundance thresholds (X-axis, log scale). Core size declines with higher abundance cutoffs, illustrating that while many taxa are shared across individuals, most occur at low relative abundance. Line color reflects prevalence (proportion of individuals harboring each taxon), with lighter shades indicating higher prevalence.

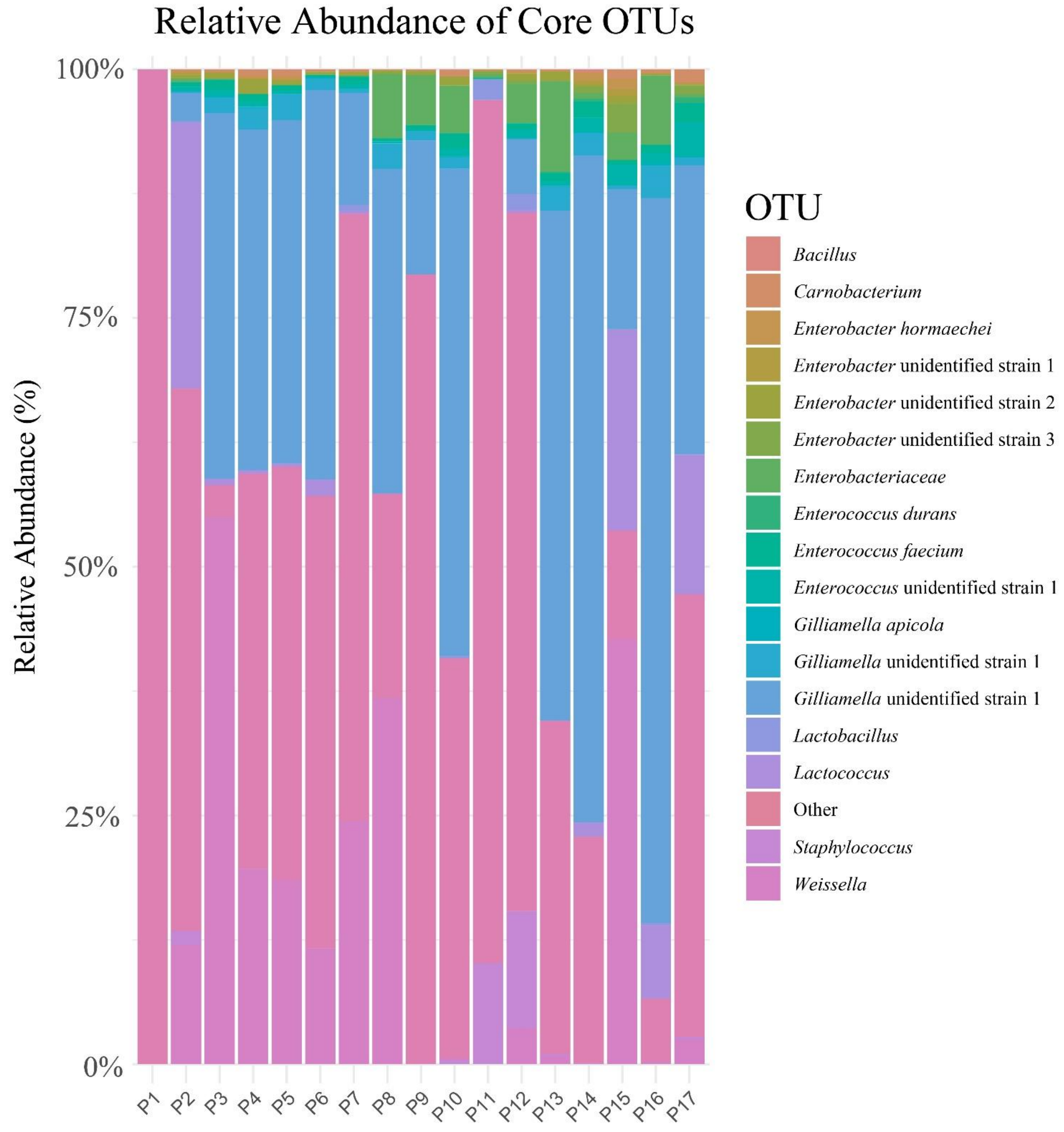


**Supplementary Figure S5**. **Stacked barplot showing the relative abundance of core OTUs in individual *Poecilus* beetles.** Each bar represents the gut microbiota composition of a single beetle, with colors indicating different core operational taxonomic units (OTUs). *Gilliamella*, *Weissella*, and *Lactococcus* dominate across samples, although their relative contributions vary markedly among individuals. Some beetles exhibit a more diverse distribution of core taxa, while others are heavily dominated by one or two genera.

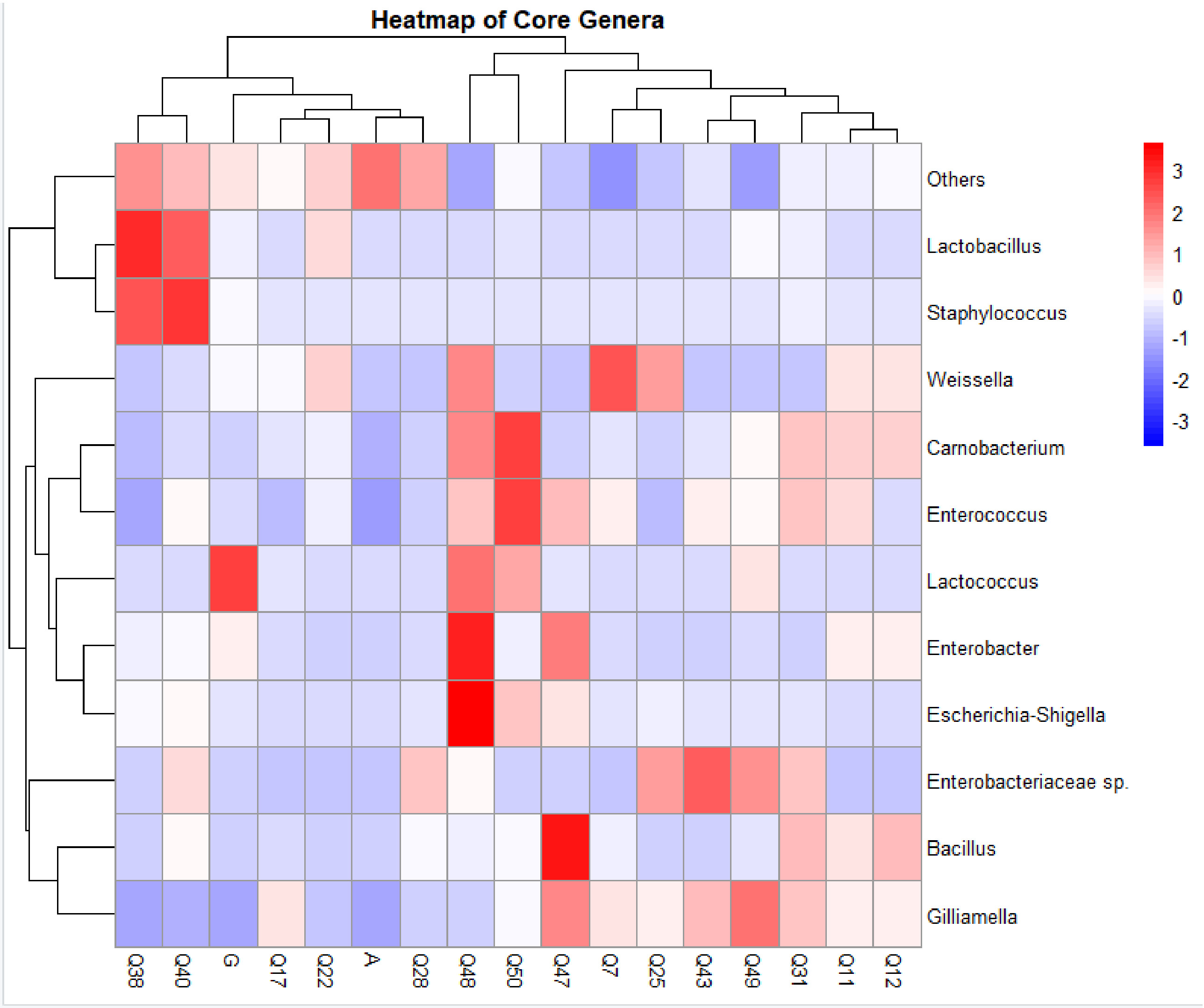


**Supplementary Figure S6**. **Heatmap showing the centered and scaled relative abundance of core bacterial genera across *Poecilus* samples.** Colour intensity represents the z-score of each genus per individual, with red indicating higher-than-average abundance and blue indicating lower-than-average levels. Clustering of both samples and genera highlights patterns of co-occurrence and individual variation in gut community structure, with notable fluctuations in *Gilliamella*, *Weissella*, and *Staphylococcus* abundance among individuals.

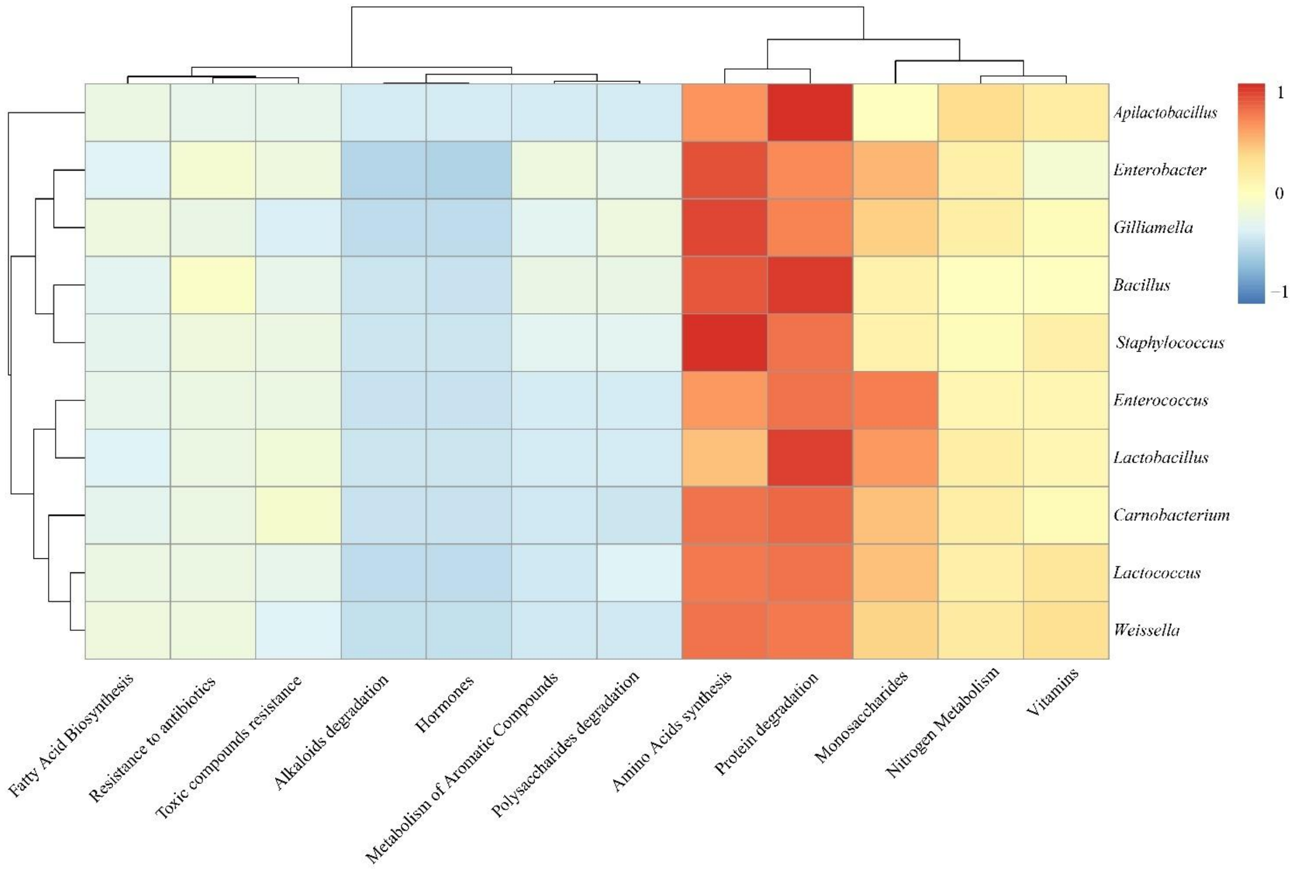


**Supplementary Figure S7.** Functionality heatmap of each core gut microbiome taxa estimated from genome analysis on KEGG and RAST SEED.

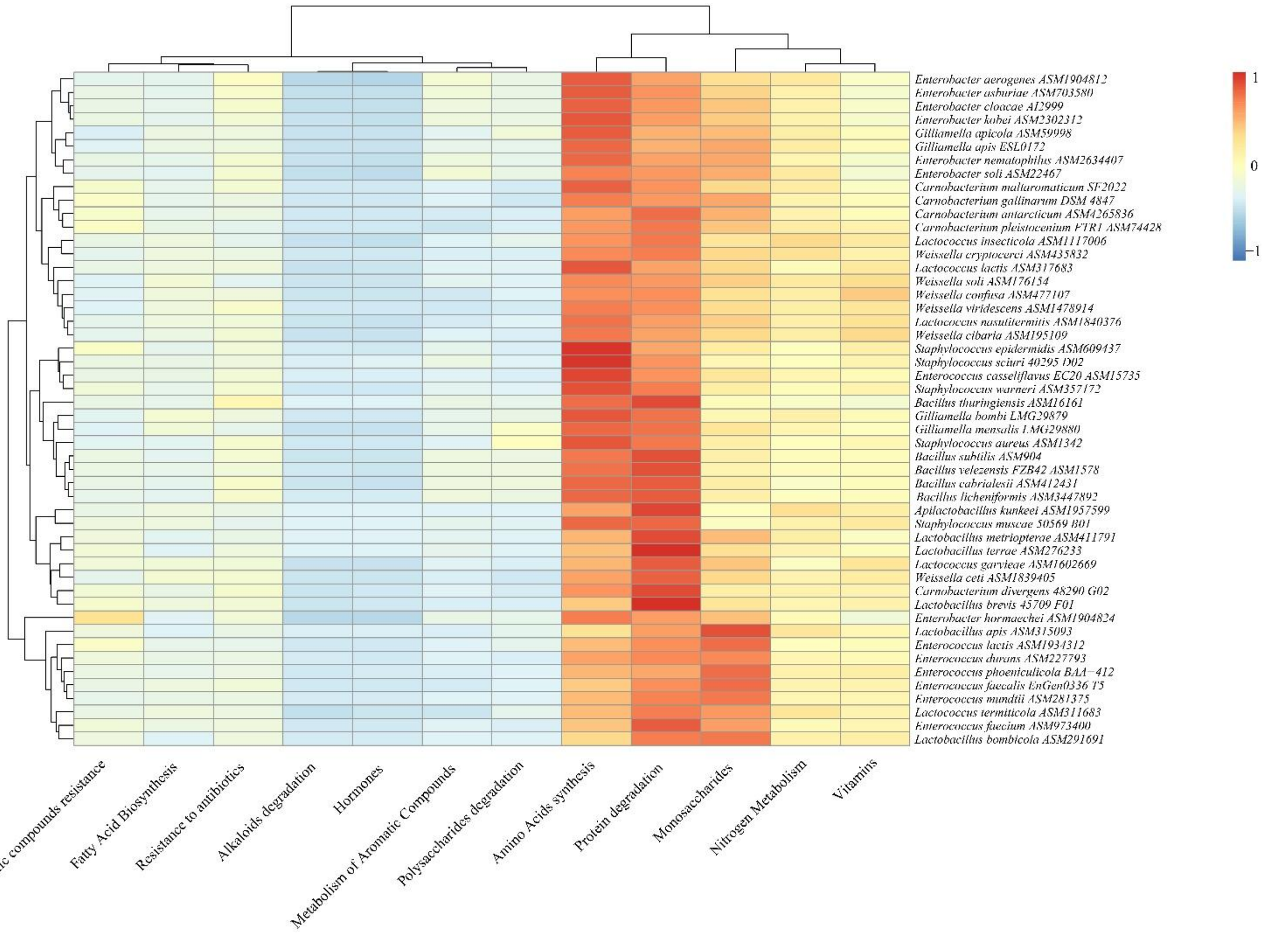


**Supplementary Figure S8.** Functionality heatmap of each core gut microbiome taxa estimated from genome analysis on KEGG and RAST SEED at species level.

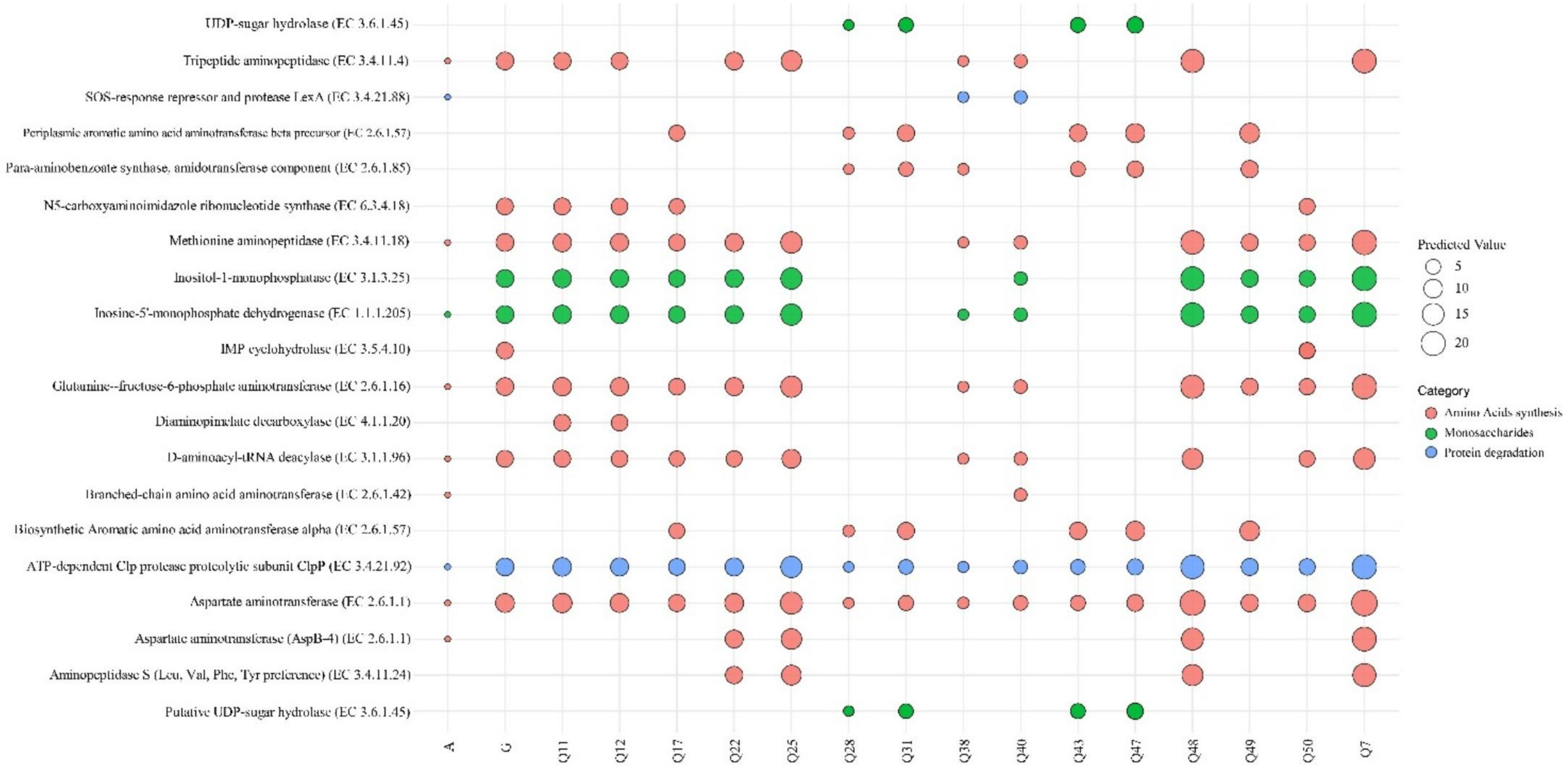


**Supplementary Figure S9.** The bubble plot shows the distribution and relative abundance of the top 10 EC numbered enzymatic pathways across all samples.

**Bibliography**

Huang, W. F., & Solter, L. F. (2013). Comparative development and tissue tropism of *Nosema apis* and *Nosema ceranae*. Journal of invertebrate pathology, 113(1), 35-41. https://doi.org/10.1016/j.jip.2013.01.001

Joyner, J., Wanless, D., Sinigalliano, C. D., Lipp, E. K. (2014). Use of quantitative real-time PCR for direct detection of Serratia marcescens in marine and other aquatic environments. A.E.M. 80, 1679–1683. https://doi.org/10.1128/AEM.02755-13

Lane, D. J. (1991). 16S/23S rRNA sequencing. Nucleic acid techniques in bacterial systematics.

Kešnerová, L., Mars, R. A., Ellegaard, K. M., Troilo, M., Sauer, U., & Engel, P. (2017). Disentangling metabolic functions of bacteria in the honey bee gut. PLoS biology, 15(12), e2003467.

Rinttilä, T., Kassinen, A., Malinen, E., Krogius, L., & Palva, A. (2004). Development of an extensive set of 16S rDNA-targeted primers for quantification of pathogenic and indigenous bacteria in faecal samples by real-time PCR. Journal of applied microbiology, 97(6), 1166-1177.

Ludwig, W., Strunk, O., Westram, R., Richter, L., Meier, H., Yadhukumar, A., ... & Schleifer, K. H. (2004). ARB: a software environment for sequence data. *Nucleic acids research*, *32*(4), 1363-1371.

Bergmark, L., Poulsen, P. H. B., Al-Soud, W. A., Norman, A., Hansen, L. H., & Sørensen, S. J. (2012). Assessment of the specificity of Burkholderia and Pseudomonas qPCR assays for detection of these genera in soil using 454 pyrosequencing. *FEMS microbiology letters*, *333*(1), 77-84.

Meeus, I., Vercruysse, V., & Smagghe, G. (2012). Molecular detection of Spiroplasma apis and Spiroplasma melliferum in bees. *Journal of Invertebrate Pathology*, *109*(1), 172-174.

# Appendix I: NGS data analysis and management

### *Pre-processing of Raw Reads and Data Filtering*

To ensure data quality and improve the accuracy of microbial community analyses, raw sequencing data underwent a structured pre-processing workflow. While traditional filtering approaches, such as abundance thresholding and prevalence-based filtering, are commonly used in microbiome research, they were found to be inadequate in this study due to their limitations in preserving biologically meaningful taxa.

Abundance threshold filtering was applied by setting OTUs with fewer than 10 reads per sample to zero. Although this method aimed to remove sequencing artifacts, it was ultimately ineffective, as it eliminated an insufficient number of low-abundance OTUs, allowing many potentially spurious taxa to remain in the dataset. Prevalence-based filtering was also tested, where OTUs were retained only if they were present in at least 50% of samples. However, this approach was found to be overly stringent, reducing the dataset from 938 OTUs to only 21 OTUs. This substantial data loss risked excluding ecologically relevant taxa, making prevalence filtering unsuitable for this dataset.

To avoid arbitrary thresholds and better capture meaningful microbial associations, Mutual Information (MI)-based filtering was implemented following the method described by Mokhtari and Ridenhour (2022). This approach applies information theory and graph theory to assess statistical dependencies between OTUs, allowing for the identification and removal of weakly connected or isolated taxa while preserving biologically relevant community structures. The MI filtering process involved computing pairwise MI values between OTUs to assess their co-occurrence patterns using the `infotheo` package in R. A microbial association network was then constructed using the `igraph` package, where nodes represented OTUs and edges represented significant MI-based connections. An optimized threshold ($\tau$) was determined based on MI retention analysis, with the final optimal threshold identified as $\tau = 0.01$. This threshold maximised network robustness while minimizing the removal of ecologically important taxa. Following this step, 210 OTUs were removed, and the final OTU table contained 327 OTUs across 17 samples, ensuring a balance between data retention and artifact removal. The total information loss after MI filtering was 4.25%, indicating that the method effectively reduced noise while preserving the dataset's biological integrity.

To confirm that this information loss was not random, bootstrap testing was performed. A p-value was computed to test whether filtering loss was greater than expected by random sampling. The low p-value ($< 0.05$) indicated that the 4.25% information loss after MI filtering was significantly lower than would be expected by random chance, demonstrating that the filtering strategy was both effective and non-random.

To assess the impact of MI-based filtering, alpha diversity (Shannon index) and beta diversity (Bray-Curtis dissimilarity, PCoA analysis) were calculated before and after filtering. Additionally, PERMANOVA (adonis2 test) was performed to determine whether MI-based filtering significantly altered microbial community composition. The effects of filtering were further analyzed through microbial association network visualization, which was conducted both before and after MI-based filtering. Network connectivity metrics were examined to evaluate how filtering influenced microbial interactions and preserved ecologically relevant taxa. By shifting from traditional threshold-based filtering to an adaptive MI-based approach, this study ensured that microbial diversity assessments reflected true biological signals rather than arbitrary filtering constraints. The combination of statistical filtering, network-based refinement, and diversity analysis resulted in a more robust and biologically meaningful microbiome dataset.

### *Defining Core Gut Microbiota*

Following the pre-processing and Mutual Information (MI)-based filtering of raw sequencing data, the resulting dataset was further refined through a multi-step normalisation and scaling procedure to ensure biologically realistic comparisons of microbial abundance across samples. Initially, raw taxon-level read counts derived from 16S rRNA gene amplicon sequencing were adjusted to account for gene copy number variation. Each taxon's read count was divided by its typical 16S rRNA gene copy number, producing copy-normalised counts and mitigating overrepresentation of taxa with multiple gene copies. These copy-normalised values were then converted into relative abundances. To obtain absolute abundance estimates, relative abundances (0–1) were multiplied by the total bacterial load per sample as measured by qPCR targeting universal eubacterial 16S rRNA genes.

The core gut microbiota of *Poecilus* spp. was subsequently defined using a combined prevalence-abundance framework, applied across multiple taxonomic ranks. At the operational taxonomic unit (OTU) level, serving

as a proxy for species resolution, core members were identified based on two conservative criteria; a prevalence threshold of ≥80% (present in at least 80% of samples) and a mean relative abundance threshold of ≥0.01% across all samples. These parameters were selected based on occupancy-abundance distributions and informed by the plot_core function within the `microbiome` R package. A total of 17 OTUs met these core criteria.

To explore higher-order taxonomic trends, core OTUs were aggregated at the genus and family levels. The relative abundances of OTUs meeting the core thresholds were summed by genus and family. Only those genera and families composed of previously defined core OTUs were retained, ensuring that higher-level taxa reflected consistent and ecologically relevant microbial constituents. The distribution of these core genera and families was subsequently visualised using bar plots and heatmaps, offering insight into conserved and dominant lineages within the *Poecilus* gut microbiota.

### *Gut Microbiome Functionality Assessment and Prediction*

The defined core gut microbial taxa from NGS of 16S rRNA analysis were considered to predict the functional profile of the gut microbial communities for each *Poecilus* individuals collected. Fully sequenced genomes of type strain bacteria taxa present in NCBI GenBank were functionally annotated with RAST (SEED Viewer version 2.0) (Aziz et al., 2008; Overbeek et al., 2013) and using KEGG orthology database (Kanehisa et al., 2004). Focus on the analysis of metabolic pathways for the i. vitamins, nitrogen, monosaccharides, hormones and aromatic compounds metabolisms; ii. polysaccharides, protein and alkaloids degradation; iii. amino acids and fatty acids biosynthesis; and iv. antibiotics and toxic compound's resistance were carried out. The specific pathways considered for each class are reported in Table S2. Each microbial strain was checked for presence/absence of the selected relevant pathways. The prediction of the microbiome functionality was carried out based on the specific microbial genera relative abundance detected by NGS sequencing for each *Poecilus* individuals. Specifically, core gut microbiome taxa relative abundance was multiplied for the estimated functional capacity for each specific genus. To predict and represent the microbiota functional distribution for each *Poecilus* specimen, the functional absolute values were converted in relative value for each microbial taxa and expressed as “Predicted score value”. Functionality

assessment and prediction were performed with RStudio (v 4.3.3) using `tidyverse` and `pheatmap` packages.
